# A normative inference approach for optimal sample sizes in decisions from experience

**DOI:** 10.3389/fpsyg.2015.01342

**Published:** 2015-09-10

**Authors:** Dirk Ostwald, Ludger Starke, Ralph Hertwig

**Affiliations:** ^1^Arbeitsbereich Computational Cognitive Neuroscience, Department of Education and Psychology, Free University BerlinBerlin, Germany; ^2^Center for Adaptive Rationality, Max Planck Institute for Human DevelopmentBerlin, Germany

**Keywords:** decisions from experience, probabilistic inference, economic decision making (EDM), uncertain decision making, optimality

## Abstract

“Decisions from experience” (DFE) refers to a body of work that emerged in research on behavioral decision making over the last decade. One of the major experimental paradigms employed to study experience-based choice is the “sampling paradigm,” which serves as a model of decision making under limited knowledge about the statistical structure of the world. In this paradigm respondents are presented with two payoff distributions, which, in contrast to standard approaches in behavioral economics, are specified not in terms of explicit outcome-probability information, but by the opportunity to sample outcomes from each distribution without economic consequences. Participants are encouraged to explore the distributions until they feel confident enough to decide from which they would prefer to draw from in a final trial involving real monetary payoffs. One commonly employed measure to characterize the behavior of participants in the sampling paradigm is the sample size, that is, the number of outcome draws which participants choose to obtain from each distribution prior to terminating sampling. A natural question that arises in this context concerns the “optimal” sample size, which could be used as a normative benchmark to evaluate human sampling behavior in DFE. In this theoretical study, we relate the DFE sampling paradigm to the classical statistical decision theoretic literature and, under a probabilistic inference assumption, evaluate optimal sample sizes for DFE. In our treatment we go beyond analytically established results by showing how the classical statistical decision theoretic framework can be used to derive optimal sample sizes under arbitrary, but numerically evaluable, constraints. Finally, we critically evaluate the value of deriving optimal sample sizes under this framework as testable predictions for the experimental study of sampling behavior in DFE.

## Introduction

“Decisions from experience” (DFE) refers to a body of work that emerged in research on behavioral decision making over the last decade. The work on DFE is held together by the central question of how humans search for information and make decisions with economic consequences in uncertain environments. Perhaps the most popular experimental paradigm used to study DFE is the “sampling paradigm” (Hertwig et al., 2004; Hertwig and Erev, 2009; Hertwig, 2012): Respondents are presented with two payoff distributions on a computer screen. A box represents each distribution, which contains a set of outcomes that occur with some probability. The participants explore each of the distributions by sampling from them. Specifically, clicking on a box triggers a random draw of an outcome from the associated set of outcomes. The participants are permitted to sample until they feel confident enough to decide which distribution is “better,” in the sense that they would prefer to draw from it during a final trial involving real monetary payoffs.

A commonly employed measure to capture the behavior of participants in the sampling paradigm is the sample size, i.e., the number of draws which participants choose to obtain from each box prior to terminating sampling. A repeated finding is that the typical sample sizes are rather low, dependent on the values of the observed outcomes (Hertwig et al., 2004; Hau et al., 2008) and modulated by the specific experimental setting (Hills and Hertwig, 2012; Lejarraga et al., 2012; Hills et al., 2013; Frey et al., 2014; Wulff et al., 2015). Given that all parameters of the DFE problem (outcomes and probabilities for each distribution) are known to the experimenter, an obvious question is how many samples a participant would draw “optimally.” The derivation of an “optimal” sample size for a given DFE problem can then be used as a normative benchmark that participants' search effort can be compared to. It can also inform quantitative theories that aim to explain why people deviate from this benchmark. Perhaps surprisingly, the question of an optimal sample size for the DFE sampling paradigm has so far not been addressed in the literature. The central aim of the current study is thus to develop such a normative benchmark by drawing on the classical literature on optimal sample sizes in probabilistic frameworks.

The notion of an “optimal” sample size is, of course, a relative concept: A given sample size can be optimal with respect to certain constraints and suboptimal with respect to others. As an example, consider a participant, whose objective is to invest as little time as possible in the experiment. For her, the optimal sample size would be zero, and the decision for a distribution that is “better” at the final draw would correspond to random guessing. Because the notion of “optimal” sample sizes is a relative concept, we have to introduce a set of assumptions or constraints that define when the benchmark applies. These assumptions may be classified into one “strong” assumption and several “weaker” assumptions.

The “strong” assumption that we make is that the problem of how much to sample can be solved by some form of statistical inference. This assumption can be stated as follows: a reasonable approach to making a sampling-based choice is to estimate the expected values of each distribution, and choosing the one deemed to offer the largest expected value. This assumption is common and implicit in many previous experimental and theoretical studies of the sampling paradigm. These studies have mostly been carried out in the context of the “description-experience gap.” The description-experience gap is the experimental finding, that choice behavior is systematically different dependent on whether information about payoffs and probabilities is learned sequentially as in DFE or stated explicitly in terms of outcomes and their associated probabilities, a paradigm referred to as “decisions from description” (DFD). As reviewed in Hertwig (2012), the “description-experience gap” may non-exclusively be explained by either of the following (1) reliance on small samples (including recency; see Hertwig et al., 2004), (2) weighting of experienced probabilities, and (3) format-dependent cognitive heuristics, all of which imply some form of statistical inference on the parameters of the underlying gamble. Specifically, the explanation of the description-experience gap by reliance on small samples states that participants' behavior is due to sampling error that renders the frequency-based estimate for the probabilities of outcomes less accurate than the explicitly stated outcome probabilities in DFD (Hertwig et al., 2004; Hau et al., 2008). Obviously, this explanation implies that participants aim to infer the true, but unknown, probabilities of the experienced outcomes. Consistent with this, it could be shown that attenuating sampling error by encouraging participants to sample more decreased the description-experience gap (Hau et al., 2008). Another approach to explain the description-experience gap has been to apply the non-linear weighting of experienced probabilities and outcomes in the evaluation of an option's value as suggested by prospect theory (Hau et al., 2008; Ungemach et al., 2009). Again, this implies that participants infer the unknown probabilities, which, upon non-linear weighting are combined with the outcome information to evaluate the option's desirability. Interestingly, in these studies it has been shown that the discrepancy between experienced outcome frequencies and the subjective estimates thereof is rather low (Fox and Hadar, 2006; Hau et al., 2008). Finally, also approaches referred to as “cognitive heuristics,” such as the “natural mean heuristic” imply a form of statistical inference (Hertwig and Pleskac, 2008). The natural mean heuristic states that participants solve the DFE problem by calculating the sample mean for each distribution and then choose that one with the larger mean. Clearly, the “natural mean heuristic” may be viewed as the psychologically plausible pendant to the estimation of an expected value by means of a sample average.

Importantly, as will be seen, the “inference assumption” renders the notion of optimal sample sizes in DFE a concept that can be addressed in the maximum expected utility (MEU) framework for statistical decisions, originally developed by Raïffa and Schlaifer (1961). It should be noted, however, that while the inference assumption in sampling DFE is widespread, it is by no means without alternatives, as we will address in more detail in the Discussion section. Notably, in our current treatment we do not claim that the individuals solve the sample-size problem by means of statistical inference. We merely note that this assumption has been made (implicitly or explicitly) previously, and thus develop a framework for an optimal sample size on the basis of this assumption.

In addition to the inference assumption, we introduce a number of “weaker” constraints or assumptions to arrive at the notion of an “optimal” sample size. These include, for example, the invocation of utility functions, sampling costs, pre-determined sample sizes, and available information about the distributions' possible outcomes. These assumptions are “weaker,” because they are ultimately consequences of the inference assumption. In other words, if we were to refute the inference assumption, these additional assumptions would presumably not enter a framework to determine optimal sample sizes, but others would in their stead. In addition, as discussed below, some of these assumptions (for example the available information about outcomes) can readily be relaxed by extending the current approach.

The outline of our manuscript is as follows. In the preceding Sections, we formalize the sampling paradigm and precisely specify the “inference assumption.” In the Section “The Maximal Expected Utility Framework”, we provide a general introduction to the optimal sample size approach by Pratt et al. (1995), referred to as the “maximal expected utility” (MEU) framework here and in the following. We further outline two variants of it that will be applied in the Sections “Optimal Sample Sizes for Parameter Point Inferences” and “Optimal Sample Sizes for Bayesian Parameter Inference,” respectively. These variants may be referred to as the “classical” and the “Bayesian” inference approaches, respectively. In the preceding Sections, we derive optimal sample sizes under relatively strong assumptions about the decision maker's representation of the problem. In the Section “Numerical Solutions” we present a numerical approach to the derivation of optimal sample sizes under the MEU framework using weaker assumptions. Finally, we discuss the potential applications of the current framework in experimental research on DFE. The Supplementary Material of this manuscript comprises additional derivations, as well as the Matlab (MathWorks, Natick, MA) code that implements the simulations and numerical schemes discussed and generates all figures shown.

In sum, the manuscript makes the following novel contributions to the literature on DFE. First and foremost, we explicitly relate the question of optimal sample sizes in the sampling paradigm to the classical literature on statistical decision theory. To this end, we provide a simplified treatment of the optimal sample size theory developed in Raïffa and Schlaifer (1961) and Pratt et al. (1995) and show how this framework can be applied to the sampling paradigm. In addition to this classical treatment, we review the derivation of optimal sample sizes under “Bayesian” inference schemes (Bernardo and Smith, 1994). In this vein, we give an explicit form of the “expected maximized expected posterior utility function” in a beta-Bernoulli scenario, which, to the best of our knowledge, has so far been absent from the works of Pratt et al. (1995) and Bernardo and Smith (1994). Further, in our application of the MEU framework to the DFE sampling paradigm, we go beyond analytically established results by showing how the framework can be used to derive optimal sample sizes under arbitrary, but numerically evaluable constraints. Finally, we critically evaluate the value of deriving testable predictions of optimal sample sizes under the MEU framework for the study of sampling behavior in DFE.

## Formalization of the DFE problem

A few preliminary remarks on the mathematical notation are in order. For simplicity, we use the applied notation for probability distributions specified in terms of probability mass or density functions. In this notation, *p*(*x*) can refer to the probability distribution, the probability density function, or the probability mass function of a random variable *x*, whichever is appropriate from the context. For sets of natural numbers (and zero), we use the following symbols: $\mathbb{N}:=\left\{1,2,\ldots \right\},\;{\mathbb{N}}^{0}:=\mathbb{N}\cup \left\{0\right\},{\mathbb{N}}_{n}:=\left\{1,2,\ldots ,n\right\},\;{\mathbb{N}}_{n}^{0}:={\mathbb{N}}_{n}\cup \left\{0\right\},\;n\in \mathbb{N}$; for the set of integers, we use ℤ. For functions, we use the standard notation *f*: *D* → *R, x* ↦ *f*(*x*): = (·), where *D* and *R* denote the domain and range of *f*, respectively, *x* ∈ *D* the function's argument, and *f*(*x*) ∈ *R* the function's value, which is defined by some closed-form expression (·). Further, we take a rather loose approach to denoting optimization operations in order to keep the notation straightforward. To this end, we denote the maximum and the minimum of a function *f* over its domain *X* by max_*x* ∈ *X*_*f*(*x*) and min_*x* ∈ *X*_*f*(*x*), respectively, and (usually) implicitly assume that they exist and are unique. Likewise, we denote the elements *x*^*max*^, *x*^*min*^ ∈ *X* that correspond to the maximum or minimum of a function *f* by $x^{max}=\arg\;\max_{x\in X}f\left(x\right)$ and $x^{min}=\arg\;\min_{x\in X}f\left(x\right)$, again, implicitly assuming that these elements exist and are unique.

The sampling paradigm in research on DFE can be conceived in the following form: A human participant is presented with two “binary payoff distributions” *p*_*G*_*A*__ and *p*_*G*_*B*__, where by “binary payoff distribution” *p*_*G*_ we understand a probability distribution of a random variable *x* taking on two possible values *x*_1_, *x*_2_ ∈ ℤ, specified by the probability mass function.

(1) $$\begin{array}{l} p_{G}\left(x=x_{1}\right)=\theta \text{ and }p_{G}\left(x=x_{2}\right)=1-\theta , \end{array}$$

and parameterized by θ ∈ [0, 1]. The expected value of a binary payoff distribution is given by:

(2) $$\begin{array}{l} E\left(x\right)=\theta x_{1}+\left(1-\theta \right)x_{2}. \end{array}$$

**Figure 1:**
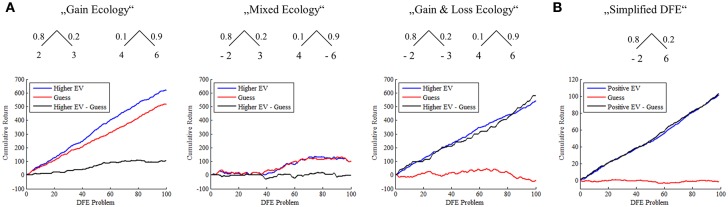
**(A)** Simulation of the superiority of a higher EV choice strategy over random choice across different DFE ecologies. The panels depict the cumulative return of two decision makers obtaining final samples from two urns according to a “higher expected value” choice rule and a random choice rule. Across different ecologies the “higher EV” choice rule outperforms the random choice rule, the amount by which depending on the specific DFE ecology. More specifically, in the “Gain Ecology” two binary payoff distributions were uniformly sampled from the space ℕ_10_×ℕ_10_×[0, 1] and “final” samples obtained from either the binary payoff distribution with the higher expected value (“higher EV”) or from either payoff distribution with equal probability (“Guess”). The cumulative returns of the “higher EV” choice rule outperform the random choice rule from approximately 40 DFE problems on in this realization. In the “Mixed Ecology,” two binary payoff distributions were uniformly sampled from {−10, −9, …, 9, 10} × {−10, −9, …, 9, 10} × [0, 1]. While the random choice rule results in approximately equal gains and loss and thus a cumulative return centered around 0, the higher EV choice rule yields cumulative gains. Finally, in the “Gain and Loss Ecology” one binary payoff distribution was sampled from ℕ_10_×ℕ_10_×[0, 1], while the other was sampled from {−10, −9, …, −1} × {−10, −9, …, −1} × [0, 1]. Again, the random choice rule results in approximately equal gains and loss, while the higher EV rule always prefers the binary payoff distribution with the positive expected value in the final choice. **(B)** Simulation of the SSP and the superiority of the positive EV choice strategy over random choice. Agreeing to sample from a single binary distribution for monetary return, when the expected value of the distribution is positive, yields positive cumulative return, randomly agreeing to sample regardless of whether the expected value is positive or is not positive yields virtually no cumulative return. Throughout panels of Figure 1 the realizations shown correspond to average cumulative returns over 1000 repetitions of the 100 DFE problem sampling and choice procedures.

We denote the parameter θ of the binary payoff distribution *p*_*G*_*A*__ by θ_*A*_ and the parameter θ of the binary payoff distribution *p*_*G*_*B*__ by θ_*B*_. After being permitted to sample from the distributions *p*_*G*_*A*__ and *p*_*G*_*B*__ *ad libitum* without economic consequences (that is, without obtaining a monetary equivalent of the sampled outcomes as return), the participant is asked to decide from which payoff distribution she would prefer to draw a single realization, the outcome of which is being returned to the participant in terms of a monetary equivalent. Assuming that participants aim to maximize their expected return over many repeats of the procedure described (with possibly differing payoff distributions), a reasonable strategy, henceforth referred to as the “inference approach,” is to choose that distribution in the final choice that is believed to offer the higher expected value (for a simulation of this strategy and its superiority to random choice, see Figure 1A). The participant's task can thus be framed as an inference problem, namely, to estimate the expected values of the distributions *p*_*G*_*A*__ and *p*_*G*_*B*__, or, in other words, their parameters θ_*A*_ and θ_*B*_. In this manuscript, we are thus concerned with the question of how many samples from each distribution the agent “optimally” draws to infer the parameters θ_*A*_ and θ_*B*_, where “optimally” is understood in a relative way with respect to some boundary conditions to which we shortly turn. Please note again that the “inference approach” to the sampling paradigm is neither exclusive nor exhaustive; alternative formalizations are possible (see Discussion), and the inference approach is merely the approach we take here.

The inference approach to the sampling paradigm may itself be addressed as a formalized decision problem in at least two ways: either (1) the participant decides on the sample size for each distribution before starting to draw samples from them, or (2) the participant decides after each obtained sample whether next to sample from distribution *p*_*G*_*A*__, to sample from distribution *p*_*G*_*B*__, or to terminate the sampling process altogether and obtain a final sample with economic consequences from either *p*_*G*_*A*__ or *p*_*G*_*B*__. The latter approach corresponds to a sequential decision problem (Powell, 2011; Wiering and Otterlo, 2012). We will be concerned with the former scenario: Specifically, we discuss a normative inference framework for and solutions to the question of how many samples to draw in order to make optimal inferences (and thus good final decisions) about the payoff distributions' expected values prior to information search.

As a first step, we simplify the problem as follows: Because we assume that the two distributions do not differ in their characteristics as specified by Equations (1) and (2), and thus the optimal sample size for each of the distributions derived will be functionally identical, we formulate a simplified problem to which we shall refer to in the following as the “simplified sampling problem” (SSP) in DFE: Assume a decision maker is faced with a binary payoff distribution *p*_*G*_ specified by a probability mass function over two outcomes *x*_1_, *x*_2_ ∈ ℤ given by:

(3) $$\begin{array}{l} p_{G}\left(x=x_{1}\right)=\theta \text{ and }p_{G}\left(x=x_{2}\right)=1-\theta , \end{array}$$

and, after being permitted to sample this distribution *ad libitum*, is asked to decide whether to draw from this distribution and obtain the resulting sampled value as monetary return or not. How many samples should the decision maker draw before making this decision and what are possible conditions that constitute “optimality” for the number of samples?

In analogy to the discussion above, we postulate that a reasonable “inference” strategy for a decision maker, aiming to maximize expected return, is to choose to draw from the distribution for a monetary return, if she believes that the expected value of the distribution is larger than zero, and not, if she believes that it is equal or smaller than zero—the former would leave the decision maker's expected cumulative return identical, the latter would decrease it. In Figure 1B we show by means of simulation that also for the SSP the inference approach is superior to randomly deciding whether to sample the binary payoff distribution for a monetary return or not.

The final decision strategy in the inference approach to the SSP is trivial. However, the question of an “optimal sample size” for inference of the expected value is not. According to Lindley (1997), this problem has been firstly addressed in a fully probabilistic manner by Raïffa and Schlaifer (1961) and their approach will, by and large, be adopted here.

## The maximal expected utility framework

In this section we review the general framework for optimal decisions under uncertainty, as formulated by Raïffa and Schlaifer (1961) and Pratt et al. (1995). In essence, this framework corresponds to a probabilistic subjective utility theory (Savage, 1972) in which the dependence of probabilistic inferences on experimentation has been made explicit. Following Lindley (1997), we will refer to this framework, when used to determine a sample size, as “maximization of expected utility” (MEU). The MEU framework has three main components: (1) the product space of four sets, (2) a utility function defined on these sets, and (3) a joint probability measure on a subset of the product space. We will first discuss each feature in turn and exemplify the general concepts with the help of the SSP before turning to the question of deriving an optimal experiment or sample size under this framework.

### Preliminaries

The MEU framework rests on the specification of the Cartesian product of four sets: a set of possible “experiments” *E*, a set of possible “experimental outcomes” *Z*, a set of “terminal acts” *A*, and a set of possible “states of the world” *S*. The order of presentation of these sets is motivated by Raïffa and Schlaifer (1961) in terms of a “game against nature”: First, the decision maker chooses a specific experiment *e* ∈ *E*; then nature determines the outcome of the experiment *z* ∈ *Z*; next, the decision maker chooses a terminal action *a* ∈ *A*, and finally nature chooses the state of the world *s* ∈ *S*. While formally helpful, this ordering may somewhat obscure the applicability of the framework to the SSP as sketched above. For the SSP, the four sets underlying the MEU framework are perhaps more intuitively apprehended as follows: for a given SSP, there is one fixed “state of the world” *s* ∈ *S*. For example, this state of the world may represent the difference in expected value between the payoff distribution and zero, the parameter θ of the distribution (which will be our approach below), or the outcome probability masses of the payoff distribution. Based on this state of the world the decision maker is able to make observations *z* ∈ *Z* that are related to the state of the world in a probabilistic manner as discussed below. The kind of outcomes the decision maker observes depend on the choice of an experiment *e* ∈ *E*. In most illustrative applications of the MEU, the experiment *e* merely defines the number of independent samples to take from the underlying process. In other words, the set of experiments may be simply understood as the set of possible sample sizes *n* ∈ ℕ_0_. An outcome *z* of an experiment *e* corresponding to a sample size *n* then corresponds to a vector of *n* independent and identically distributed observations from the underlying process. For the SSP, the experiment *e* may thus correspond to the sample size taken from the binary payoff distribution, whereas an outcome *z* corresponds to the concatenated vector of *n* “observations” or “realizations” obtained on each single draw. Finally, the terminal action *a* ∈ *A* may correspond to the decision for a specific estimate of the underlying state *s* ∈ *S*. Notably, this approach addresses statistical inference as a decision problem, which corresponds to the standard way of comparing different estimation procedures (Lehmann and Casella, 1998). In the application to the SSP, the terminal action *a* ∈ *A* should not be confused with “the final choice with economic consequences,” which in our formulation resides outside of the MEU framework and is conceived as a deterministic consequence of the MEU inference approach. We denote the Cartesian product space of the relevant sets by *A* × *S* × *E* × *Z*.

The second ingredient of the MEU framework is a “utility function” defined on this Cartesian product space:

(4) $$\begin{array}{l} u:A\times S\times E\times Z\to \mathbb{R},\left(a,s,e,z\right)\mapsto u\left(a,s,e,z\right). \end{array}$$

Formally, Pratt et al. (1995) distinguish between “utility” and “value” functions, and formulate most of the theory in terms of value functions, which are conceived as utility functions reformulated with the help of a monetary equivalent or another “numeraire.” However, as the notion of utility functions appears to be more widely used (Raïffa and Schlaifer, 1961; Bernardo and Smith, 1994; Bernardo, 1997; Lindley, 1997) with respect to both the MEU framework and the general decision theoretic literature, we will refer to *u* as “utility” function and implicitly assume that it has the necessary mathematical properties of a function that sensibly represents a set of preference relations. In the MEU framework, a utility function is usually assumed to be additively decomposable into a “terminal utility” *u*_*t*_ determined by the chosen action *a* and the state of the world *s*, and an “experimentation (or sampling) utility” *u*_*s*_ determined by the experiment *e* and its outcome *z*, i.e.

(5) $$\begin{array}{r} u:A\times S\times E\times Z\to \mathbb{R},\left(a,s,e,z\right)\mapsto u\left(a,s,e,z\right)\\ :=u_{t}\left(a,s\right)+u_{s}\left(e,z\right). \end{array}$$

As it is perhaps more natural to associate a “cost” rather than a “utility” with experimentation, the MEU framework specifies the cost *c*_*s*_ of experimentation as the negative of the utility of experimentation or sampling:

(6) $$\begin{array}{l} u_{s}\left(e,z\right)=:-c_{s}\left(e,z\right). \end{array}$$

Note that we have used the subscript *s* for the utility or cost of experimentation, as it will refer to the utility or cost of “sampling” in most of the remainder.

Finally, the MEU framework assumes that the decision maker can specify a probability measure on the space *S* × *Z* for (and thus dependent on) each experiment *e* ∈ *E*. We will denote this probability measure as *p*_*e*_(*s, z*), making the dependence on the experiment (and in its application to the SSP, the sample size) explicit. The joint probability measure *p*_*e*_(*s, z*) induces the usual marginal and conditional probability measures, for which we introduce the following connotations.

1. The decision maker's “prior probability” over states of the world *p*(*s*).
2. The decision maker's marginal probability of experimental outcomes *p*_*e*_(*z*).
3. The decision maker's “likelihood” of experimental outcomes *p*_*e*_(*z*|*s*).
4. The decision maker's posterior probability over states of the world *p*_*e*_(*s*|*z*).

### The optimal experiment

Based on the preliminaries outlined above the optimal experiment in the MEU framework is given by:

(7) $$\begin{array}{l} e^{opt}:=\arg\;\max_{e\in E}\left(\underset{Z}{\int }p_{e}\left(z\right)\max_{a\in A}\left(\underset{S}{\int }p_{e}\left(s|z\right)u\left(a,s,e,z\right)ds\right)dz\right). \end{array}$$

In order to make expression (7) transparent, we first unpack it into a sequence of four steps and then apply it to the SSP. The four steps implied by Equation (7) correspond to alternating expectation and maximization operations. In the first step the expectation of the utility function *u*(*e, z, a, s*) is evaluated under the experiment-dependent posterior distribution over states of the world *p*_*e*_(*s*|*z*). The resulting quantity is a function of the experiment, the terminal decision *a*, and the experimental outcome *z* and may be referred to as the “expected posterior utility” function:

(8) $$\begin{array}{r} f:E\times Z\times A\to \mathbb{R},\left(e,z,a\right)\mapsto f\left(e,z,a\right)\\ :=\underset{S}{\int }p_{e}\left(s|z\right)u\left(a,s,e,z\right)ds. \end{array}$$

In the second step, the function *f* is maximized with respect to the action *a* ∈ *A*. This step results in the definition of a function that may be referred to as the “maximized expected posterior utility function”:

(9) $$\begin{array}{r} g:E\times Z\to \mathbb{R},\left(e,z\right)\mapsto g\left(e,z\right)\\ :=\max_{a\in A}f\left(a,e,z\right). \end{array}$$

The argument maximizing the function *g* for a given combination of *e* and *z* is referred to as the “optimal posterior act” and will be denoted by *a*^*opt*^. In the third step, the function *g* is integrated with respect to the marginal measure of the experimental outcomes *p*_*e*_(*z*), defining a third function, which may be referred to as the “expected maximized expected posterior utility” function:

(10) $$\begin{array}{l} h:E\to \mathbb{R},e\mapsto h\left(e\right):=\underset{Z}{\int }p_{e}\left(z\right)g\left(e,z\right)dz. \end{array}$$

Finally, in the fourth step the experiment *e*^*opt*^ which maximizes the function *h* is found and corresponds to the optimal experiment, i.e., that experiment, which maximizes the expected maximized expected posterior utility, or “expected utility” for short.

By introducing additional assumptions about the utility function and the nature of the space of experiments *E*, the definition of the optimal experiment in (7) can be rewritten in a way that allows for a more convenient application to the SSP. First, as above, we assume that the utility function is additively decomposable into a terminal utility *u*_*t*_ and a cost of sampling *c*_*s*_. In lieu of Equation (8) this additional assumption yields the special case:

(11) $$\begin{array}{lll} f\left(e,z,a\right) & := & \underset{S}{\int }p_{e}\left(s|z\right)\left(u_{t}\left(a,s\right)-c_{s}\left(e,z\right)\right)ds\\ = & \underset{S}{\int }p_{e}\left(s|z\right)u_{t}\left(a,s\right)ds-c_{s}\left(e,z\right), \end{array}$$

where the integral term is referred to as the “posterior expected terminal utility.” Consequently, in the special case of (11) the function *h* takes the form:

(12) $$\begin{array}{l} h\left(e\right):=\underset{Z}{\int }p_{e}\left(z\right)\max_{a\in A}\left(\underset{S}{\int }\left({p_{e}\left(s|z\right)u}_{t}\left(a,s\right)ds-c_{s}\left(e,z\right)\right)\right)dz. \end{array}$$

Secondly, we assume that the experiments *e* ∈ *E* are fully specified in terms of their sample size, that is, *E*: = ℕ_0_, *e*: = *n* ∈ ℕ_0_, and that the cost of sampling is independent of the experimental outcome *z* and linear in the sample size *n* with a proportionality constant *c*> 0. Formally, we thus have for the sampling cost *c*_*s*_:

(13) $$\begin{array}{l} c_{s}\left(e,z\right)=c_{s}\left(e\right)=c_{s}\left(n\right):=cn\;\left(c\in \mathbb{R}\right). \end{array}$$

Based on these assumptions, we can re-express (7) as specifying an optimal sample size, which we denote by *n*^*opt*^, by:

(14) $$\begin{array}{l} n^{opt}:=\arg\;\max_{n\in {\mathbb{N}}^{0}}\left(\underset{Z}{\int }p_{n}\left(z\right)\max_{a\in A}\left(\underset{S}{\int }p_{n}\left(s|z\right)u_{t}\left(a,s\right)ds\right)dz-cn\right) \end{array}$$

In the MEU framework, the argument of the argmax() operator is also referred to as the “expected net utility of sampling,” the first term of the sum of this argument is referred to as the “expected utility of sampling information” and the last term as the “sampling cost.”

**Figure 2:**
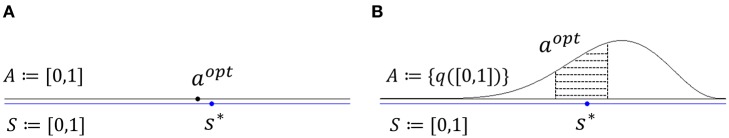
**(A)** State and action space scenario considered in the Section “Optimal Sample Sizes for Parameter Point Inference.” In this Section we consider the case that the decision maker aims to obtain an optimal sample size under the MEU inferential approach while deriving “classical point parameter estimates” of the true, but unknown, state s^*^ of the SSP. Specifically, as will become clear below, the state space *S* in Section The Maximal Expected Utility Framework corresponds to the interval [0, 1] and so does the action space *A*. **(B)** State and action space scenario considered in the Section “Optimal Sample Sizes for Bayesian Parameter Inference.” In this Section we consider the same state space, but a different action space as compared to the Section “Optimal Sample Sizes for Parameter Point Inference.” Specifically, while the state space again corresponds to the interval [0, 1], the action space now corresponds to the set of probability distributions over the interval [0, 1] and the optimal action to a member of this set. This corresponds to a decision maker that aims to obtain an optimal sample size under the MEU inferential approach while performing “Bayesian inference” about the true, but unknown, state of world.

Having established the general MEU framework and a first specialization of it in Equation (14) as basis for its application to the SSP, we are now in the position to make this application more concrete. In the Sections “Optimal Sample Sizes for Parameter Point Inference” and “Optimal Sample Sizes for Bayesian Parameter Inference,” we will illustrate two formulations of the inference approach to optimal sample sizes in DFE that differ in their notions of “inferences,” or, in general MEU terms, their notions of “actions.” Specifically, in the Sections “Optimal Sample Sizes for Parameter Point Inference” and “Optimal Sample Sizes for Quadratic Terminal Loss and Beta Prior,” we conceive the action space as a space of point estimates for the true, but unknown, state of the SSP. In the Section “Optimal Sample Sizes for Bayesian Parameter Inference,” we conceive the action space as a space of probability distributions over the states of the world. Informally, these approaches may be conceived as different types of inferences the optimizing decision maker performs as actions: “classical point parameter estimation” in the former, and “Bayesian inference” in the latter case (see Figure 2). Their parallel development illustrates the degrees of freedom in model development that exists even under a single formalized approach.

## Optimal sample sizes for parameter point inference

Based on the developments in Section Formalization of the DFE Problem, we are in the position to derive the optimal sample size as defined by the MEU framework in Equation (14). To recapitulate, we assumed that faced with a binary payoff distribution, the optimizing decision maker uses an inference approach to determine the expected value of the distribution, with the aim of choosing to sample from this distribution in a final draw with economic consequences if and only if the expected value is larger than zero. Second, we assumed that prior to entering the sampling stage, the decision maker evaluates the optimal sample size to take from the distribution. To apply the MEU framework to the SSP, we take advantage of the following simple idea: We identify the SSP with the well-studied Bernoulli distribution parameter estimation problem, by adopting the coding scheme *x*_1_: = 1 (“success”) and *x*_2_: = 0 (“fail”) for the binary payoff distribution outcomes. The assumption of determining the optimal sample size a priori then becomes a Binomial sampling problem (as opposed to Pascal sampling approaches). In this scenario, if the decision maker has committed to an estimate of the Bernoulli distribution parameter, he or she may evaluate the expected value of the distribution by means of Equation (2) and thus proceed to the final decision on whether to sample the distribution for a monetary return, or not. Note that this approach assumes that the outcomes of the binary payoff distribution are, by one means or another, known to the decision maker, an issue we will return to in the Discussion. Based on this idea and under the additional assumption of parameter point inference, the definition of the remaining components of the MEU framework for application to the SSP is straightforward:

We define the set of possible states of the world, that is, the set of possible true, but unknown, values of the underlying Bernoulli parameter, by *S*: = [0, 1]. We denote the elements of this set by θ for coherence with the standard notation of Bernoulli distributions. Likewise, we define the set of possible acts, i.e., point estimates $\hat{\theta }$ of θ, as *A*: = [0, 1]. For the Binomial sampling problem, the set of possible experiments *E* is uniquely defined by the numbers of samples *n* ∈ ℕ^0^ taken for each experiment *e* ∈ *E*. We thus identify *E*: = ℕ_0_ and use *n* to denote an experiment. Because in the Binomial sampling problem the number of observed “successes” together with the total number of observations is a sufficient statistic for the parameter of the underlying Bernoulli process, we replace the experimental outcome space {0, 1}^*n*^ by the sufficient statistic outcome space ${\mathbb{N}}_{n}^{0}$ and denote by $r_{n}\in {\mathbb{N}}_{n}^{0}$ the number of successes in a given sample:

(15) $$\begin{array}{l} r_{n}:=\overset{n}{\underset{i=1}{\sum }}z_{i}\in {\mathbb{N}}_{n}^{0},\text{ where }z:={\left(z_{1},\ldots ,z_{n}\right)}^{T}\in {\left\{0,1\right\}}^{n}. \end{array}$$

For a formal discussion of the notion of “sufficient statistics” in the MEU setting, see Section 2.2 in Raïffa and Schlaifer (1961). Intuitively, no information inherent in a sample of size *n* that is relevant for the parameters of the underlying probabilistic model is lost when recording the number of 1's *r*_*n*_ and the total number of samples *n*, rather than the complete sequences of ones and zeros. In summary, we define the problem space as:

(16) $$\begin{array}{l} A\times S\times E\times Z:=\left[0,1\right]\times \left[0,1\right]\times {\mathbb{N}}^{0}\times {\mathbb{N}}_{n}^{0}. \end{array}$$

On the problem space (16) we define a utility function:

(17) $$\begin{array}{l} u:\left[0,1\right]\times \left[0,1\right]\times {\mathbb{N}}^{0}\times {\mathbb{N}}_{n}^{0}\to \mathbb{R},\left(\hat{\theta },\theta ,n,r_{n}\right)\mapsto u\left(\hat{\theta },\theta ,n,r_{n}\right), \end{array}$$

and, as above, assume that this utility function decomposes additively into a contribution of terminal action $u_{t}\left(\hat{\theta },\theta \right)$ and a cost of experimentation or sampling *c*_*s*_(*n, r*_*n*_):

(18) $$\begin{array}{lll} u:\left[0,1\right]\times \left[0,1\right]\times {\mathbb{N}}^{0}\times {\mathbb{N}}_{n}^{0} & \to & \mathbb{R},\left(\hat{\theta },\theta ,n,r_{n}\right)\mapsto u\left(\hat{\theta },\theta ,n,r_{n}\right)\\ := & u_{t}\left(\hat{\theta },\theta \right)-c_{s}\left(n,r_{n}\right). \end{array}$$

Below, we will choose a simple, familiar form for the terminal utility. As in (13) we set

(19) $$\begin{array}{l} c_{s}:{\mathbb{N}}^{0}\times {\mathbb{N}}_{n}^{0}\to \mathbb{R},\left(n,r_{n}\right)\mapsto c_{s}\left(n,r_{n}\right):=cn \end{array}$$

for *c*> 0. Finally, we denote the experiment-dependent probability measure on the space of possible true states of the world and experimental outcomes by:

(20) $$\begin{array}{l} p_{n}\left(\theta ,r_{n}\right)=p\left(\theta \right)p_{n}\left(r_{n}|\theta \right). \end{array}$$

In the following, we will choose a simple parameterized distribution for the marginal distribution *p*(θ). For the “likelihood distribution” *p*_*n*_(*r*_*n*_|θ), we choose the Binomial distribution throughout the remainder of this study. The Binomial distribution specifies the probability of *r*_*n*_ observations of 1's (or “successes”) in an independent and identical Bernoulli sampling sequence of length *n*. We denote the binomial distribution on {0, 1, …, *n*} by the probability mass function with parameters *n* ∈ ℕ and θ ∈ [0, 1] by:

(21) $$\begin{array}{l} p_{n}\left(r_{n}|\theta \right):=Bi\left(r_{n};n,\theta \right):=\left(\begin{array}{l} n\\ r_{n} \end{array}\right)\theta^{r_{n}}{\left(1-\theta \right)}^{n-r_{n}}, \end{array}$$

where the first product term denotes the binomial coefficient in *n* and *r*_*n*_.

### Optimal sample sizes for quadratic terminal loss and beta prior

Based on the definitions (15), (16), (18), (19), and (21), we now explore optimal sample sizes for point parameter inference in SSP in a setting that allows for the analytical derivation of optimal sample sizes as a function of the prior distribution *p*(θ). To this end, we proceed as follows: we first establish that instead of a terminal utility function $u_{t}\left(\hat{\theta },\theta \right)$ as in (18), we may equally frame the problem using a “terminal opportunity loss function” $l_{t}\left(\hat{\theta },\theta \right)$. We then choose the quadratic loss function as a familiar example of an opportunity loss function. Finally, we define a parameterized probability distribution for the marginal distribution *p*(θ) and evaluate optimal sample sizes.

As discussed in Pratt et al. (1995) the MEU framework allows for the specification of the statistical decision problem in terms of “loss functions” (as familiar from classical statistics) in lieu of terminal utility functions. Notably, in the MEU framework “loss” is always understood as “terminal opportunity loss.” This means “loss” is defined as the difference between the utility that the decision maker actually realizes by choosing action *a* ∈ *A*, if the true state of the world is *s* ∈ *S*, and the greater value that the decision maker “had the opportunity” of realizing by choosing the optimal action for state *s*, denoted by $a_{s}^{opt}\in A$ without experimentation, i.e., as expected under the prior distribution over states. In other words, a terminal utility function is expressed in terms of a terminal opportunity loss function:

(22) $$\begin{array}{lll} u_{t}^{l}:A\times S & \to & \mathbb{R},\left(a,s\right)\mapsto u_{t}^{l}\left(a,s\right)\\ := & -l_{t}\left(a,s\right)+\min_{a\in A}\left(\int p\left(s\right)l_{t}\left(a,s\right)ds\right). \end{array}$$

Importantly, this definition is going beyond standard definitions of loss as merely “negative terminal utility.” However, the integral term above is independent of *a* and *s* thus corresponds to an additive constant. If considered as a function of action *a* ∈ *A*, we may thus conceive the decision maker to either maximize its expected terminal utility or minimize its expected terminal loss *l*_*t*_(*a, s*) (while minimizing expected sampling cost). The solution in terms of the optimal action is identical. The expected opportunity loss associated with a given act, however, corresponds to the negative of the expected terminal utility associated with the same act shifted by an additive constant, which is independent of *a*. If the decision maker's goal is merely to maximize the expected utility function, she may, equivalently, find the action *a* ∈ *A* that minimizes the expected terminal opportunity loss. To determine the value of the expected terminal utility function based on the minimization of the expected terminal opportunity loss function, however, the decision maker requires the evaluation of the second term in (22). Put succinctly: if the decision maker is merely interested in choosing the optimal action, it may formulate the problem either in terms of terminal opportunity loss or in terms of terminal utility. Only if, in addition, the decision maker is interested in the associated utility of the optimal act, the decision maker is required to formulate the problem in terms of a utility function.

A different perspective on the additive constant in (22) is afforded by considering the terminal utility of no experimentation. If the decision maker chooses not to experiment at all, i.e., the sample size is *n* = 0, the posterior distribution *p*_*e*_(*s*|*z*) will equal the prior distribution *p*(*s*) and Equation (12) would require the integration of the $u_{t}^{l}\left(a,s\right)$ under the prior distribution and maximization with respect *a*. This corresponds to the integration of the first term in (22) under the prior distribution and its maximization. To ensure that the utility of a sample size of *n* = 0 is zero, this value is then subtracted in the form of the second term in (22). Because of this normalization property, and because we have introduced the MEU framework in terms of a terminal utility function, which is to be maximized, we will maintain this convention and explicitly take the second term in (22) into account.

The specific loss function we use in the remainder of this Section is the “quadratic loss function,” a classically chosen loss function for point estimation problems (Lehmann and Casella, 1998). In (22), we thus replace *l*_*t*_(*a, s*) by $l_{t}\left(\hat{\theta },\theta \right)$ and define:

(23) $$\begin{array}{l} l_{t}:\left[0,1\right]\times \left[0,1\right]\to {\mathbb{R}}_{+},\left(\hat{\theta },\theta \right)\mapsto {\left(\theta -\hat{\theta }\right)}^{2}. \end{array}$$

The quadratic terminal loss function is zero, if the chosen act, i.e., point estimate $\hat{\theta }$, equals the true, but unknown, state value θ, and the square penalizes large deviations from the true, but unknown, value more strongly than small deviations. Summarizing (22) and (23), we thus define the following terminal utility function in the current section.

(24) $$\begin{array}{lll} u_{t}^{l}:\left[0,1\right]\times \left[0,1\right] & \to & \mathbb{R},\left(\hat{\theta },\theta \right)\mapsto u_{t}^{l}\left(\hat{\theta },\theta \right)\\ := & -{\left(\theta -\hat{\theta }\right)}^{2}+\min_{\hat{\theta }\in \left[0,1\right]}\left(\int p\left(\theta \right){\left(\theta -\hat{\theta }\right)}^{2}d\theta \right),\; \end{array}$$

which can be substituted in Equation (18). The total utility incurred by the decision maker then comprises a contribution of terminal opportunity loss, a contribution of sampling costs, and a fixed offset, which depends on the prior distribution of θ.

To be able to derive optimal sample sizes, we further have to specify the form of the prior distribution *p*(θ) in (24). In this section, we assume that the decision maker is able to quantify its initial uncertainty with respect to the parameter θ by means of a beta distribution, i.e., a distribution on the interval [0, 1] characterized by the two parameter probability density function:

(25) $$\begin{array}{l} p\left(\theta \right):=Be\left(\theta ;\alpha ,\beta \right):=\frac{\Gamma \left(\alpha +\beta \right)}{\Gamma \left(\alpha \right)\Gamma \left(\beta \right)}\theta^{\alpha -1}{\left(1-\theta \right)}^{\beta -1}, \end{array}$$

where

(26) $$\begin{array}{l} \Gamma :{\mathbb{R}}_{+}\to \mathbb{R},x:=\Gamma \left(x\right)=\overset{\infty }{\underset{0}{\int }}t^{x-1}\exp\left(-t\right)dt, \end{array}$$

denotes the Gamma function and α, β > 0 are the parameters (which may be interpreted as the number of “virtual prior observations” of 1's and 0's, respectively). The beta prior distribution in the Binomial sampling scenario has well-known advantages: It is specified on the parameter space of interest for Binomial sampling, it is the conjugate-prior distribution for the Binomial distribution, i.e., the data conditional (posterior) distribution is again a Beta distribution with well-known and simple formulas for its parameters, and it allows for the specification of an “uninformative” prior distribution in the “reference distribution” sense (Bernardo and Smith, 1994) by choosing α = β = 0.5. In summary, we thus specify the following joint probability distribution over states of the world θ and experimental outcomes *r*_*n*_.

(27) $$\begin{array}{l} p_{n}\left(\theta ,r_{n}\right)=p\left(\theta \right)p_{n}\left(r_{n}|\theta \right):=Be\left(\theta ;\alpha ,\beta \right)Bi\left(r_{n};n,\theta \right). \end{array}$$

**Figure 3:**
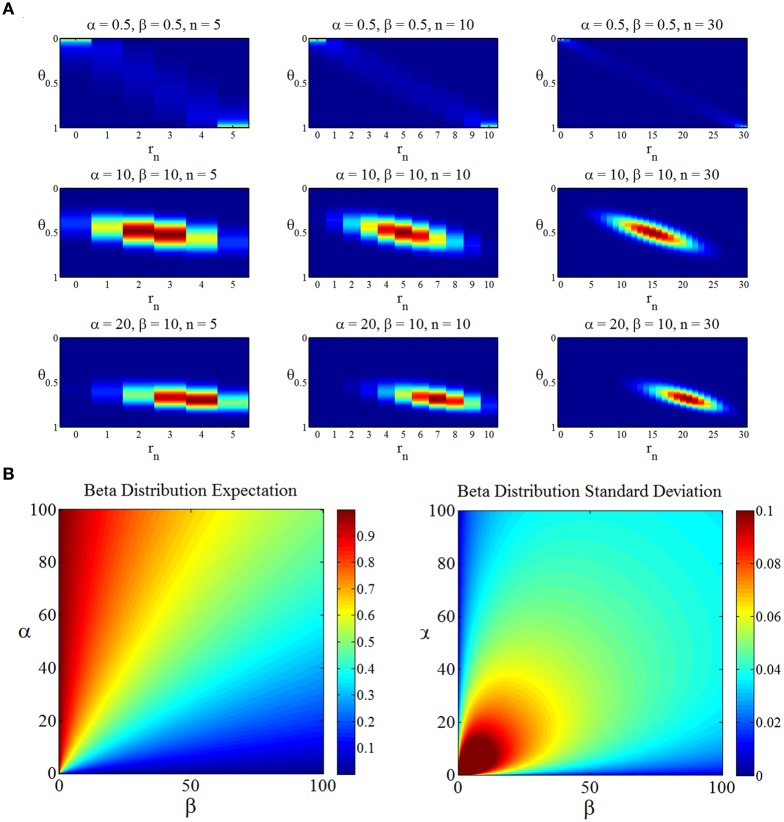
**(A)** The joint distribution over “states of the world” θ and “experimental outcomes” *r*_*n*_ as a function of the prior distribution parameters α, β (rows) and the sample size *n* (columns). The first row depicts the joint distribution for the reference prior parameter settings, the second row for a prior distribution centered on 0.5 with higher implied certainty, and the last row for prior distribution parameters reflecting a biased assumption about the state of the world θ. **(B)** Dependence of the first two central moments of the beta distribution on its parameters. In general, if both α and β increase, the uncertainty implied by the beta distribution decreases.

Notably, this joint distribution is dependent on the sample size *n* and the beta prior parameters α and β. We visualize this joint density for selected values of *n* and α, β in Figure 3A. Additionally, we depict the dependence of the beta distribution expectation and standard deviation on its parameters in Figure 3B.

Based on the definitions (24) and (25), we may now evaluate optimal sample sizes as a function of the prior parameters α, β and the value of the sampling cost proportionality constant *c* > 0 by means of the general definition of the optimal experiment in the MEU framework (Equation 14).

(28) $$\begin{array}{l} n^{opt}=\arg\underset{{}_{n\in {\mathbb{N}}_{0}}}{\min}(\int_{\left\{0,1,\ldots ,n\right\}}p_{n}(r_{n})\underset{{}_{\hat{\theta }\;\in \;[0,1]}}{\min}\\ \;(\int_{\left[0,1\right]}p_{n}\left(\theta |r_{n}\right)(l_{t}\left(\hat{\theta },\theta \right)\\ \;-\underset{{}_{\hat{\theta }\;\in \;\left[0,1\right]}}{\min}\int_{\left[0,1\right]}p\left(\theta \right)l_{t}\left(\hat{\theta },\theta \right)d\theta )d\theta )dr_{n}+cn). \end{array}$$

Note that we have exchanged maximization with minimization for simplicity. Because the additive terminal opportunity loss constant is independent of θ and *r*_*n*_, we may equivalenty write (28) as:

(29) $$\begin{array}{l} n^{opt}=\arg\underset{{}_{n\;\in \;{\mathbb{N}}_{0}}}{\min}(\int_{\left\{0,1,\ldots ,n\right\}}p_{n}(r_{n})\underset{\hat{\theta }\;\in \;[0,1]}{\min}\\ \;\left(\int_{\left[0,1\right]}p_{n}\left(\theta |r_{n}\right)l_{t}\left(\hat{\theta },\theta \right)d\theta \right)dr_{n}\\ \;-\underset{{}_{\hat{\theta }\in \left[0,1\right]}}{\min}\int_{\left[0,1\right]}p\left(\theta \right)l_{t}\left(\hat{\theta },\theta \right)d\theta +cn). \end{array}$$

As shown by Pratt et al. (1995), the evaluation of (29) can be performed analytically, as we sketch in the following.

We first derive the probability distributions involved based on the specification of *p*_*n*_(θ, *r*_*n*_) in (27). To this end, the conditional distribution of θ given *r*_*n*_ is well-known to conform to an updated beta distribution.

(30) $$\begin{array}{l} p_{n}\left(\theta |r_{n}\right)=Be\left(\theta ;\alpha +r_{n},\beta +n-r_{n}\right). \end{array}$$

Perhaps less well-known is the marginal distribution of experimental outcomes under the assumptions of a marginal beta distribution over θ and a Binomial conditional distribution over *r*_*n*_, i.e., the parametric form of:

(31) $$\begin{array}{l} p_{n}\left(r_{n}\right)=\overset{1}{\underset{0}{\int }}Bi\left(x;\theta ,n\right)Be\left(\theta ;\alpha ,\beta \right)d\theta . \end{array}$$

This distribution is given by a binomial-beta distribution (also referred to as “hyperbinomial distribution”) with probability density function (e.g., Bernardo and Smith, 1994):

(32) $$\begin{array}{lll} Bb\left(r_{n};\alpha ,\beta ,n\right) & = & \frac{\Gamma \left(\alpha +\beta \right)}{\Gamma \left(\alpha \right)\Gamma \left(\beta \right)\Gamma \left(\alpha +\beta +n\right)}\left(\begin{array}{l} n\\ r_{n} \end{array}\right)\\ \;\Gamma \left(\alpha +r_{n}\right)\Gamma \left(\beta +n-r_{n}\right). \end{array}$$

for *r*_*n*_ = 0, 1, …, *n* and *n* = 1, 2, …  with *n* ≥ *k*.

Based on (30) one finds that the inner integral expression in the first term of (29) evaluates to:

(33) $$\begin{array}{rcl} \underset{\left[0,1\right]}{\int }l_{t}\left(\hat{\theta },\theta \right)p_{n}\left(\theta |r_{n}\right)d\theta & = & \frac{\left(\alpha +r_{n}\right)\left(\beta +n-r_{n}\right)}{{\left(\alpha +\beta +n\right)}^{2}\left(\alpha +\beta +n+1\right)}\\ +{\left(\frac{\alpha +r_{n}}{\alpha +\beta +n}-\hat{\theta }\right)}^{2}. \end{array}$$

which takes on its minimal value with respect to $\hat{\theta }$ for:

(34) $$\begin{array}{l} {\hat{\theta }}_{\alpha ,\beta ,n,r_{n}}^{opt}:=\frac{\alpha +r_{n}}{\alpha +\beta +n}. \end{array}$$

Please see the Supplementary Material for proofs of (33) and (34).

**Figure 4:**
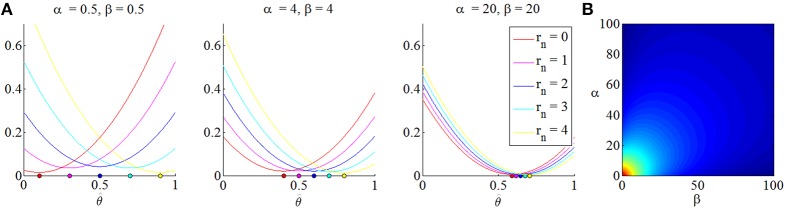
**(A)** Posterior terminal opportunity loss as a function of possible acts or point estimates $\hat{\theta }$. For a sample size *n* = 4 and prior distribution parameters noted above each subpanel, the three panels depict the posterior terminal opportunity loss for all possible values of the sufficient statistic *r*_*n*_ and their respective minimal point. The higher the prior certainty (i.e., the lower the variance of the prior beta distribution), the less dependent is the location of the posterior terminal opportunity loss on the point parameter estimate. **(B)** For a sample size of *n* = 16 and an observation of *r*_*n*_ = 8, this figure depicts the posterior terminal opportunity loss at its minimum. Notably, the minimized posterior terminal opportunity loss is symmetric in the prior parameters and decreases with higher prior certainty. Note that the posterior terminal opportunity loss is a function of the experimental outcome.

The quantity (33) is referred to as “posterior terminal opportunity loss” and, for a given setting of the prior parameters, is a function of the sample size *n*, the point estimate or “act” $\hat{\theta }$ and the experimental outcome *r*_*n*_. Its minimizer ${\hat{\theta }}_{\alpha ,\beta ,n,r_{n}}^{opt}$ is referred to as “optimal posterior act” and here assumes the meaning of an optimal point estimate for θ. Notably, ${\hat{\theta }}_{\alpha ,\beta ,n,r_{n}}^{opt}$ is dependent on the prior distribution parameters, the sample size and the observed outcome *r*_*n*_. Interestingly, in contrast to estimators derived in the standard framework of classical statistics and their associated optimality theory, by means of the MEU framework, this estimator has already been established as “optimal” for every possible state of the world. In Figure 4 we visualize the posterior terminal opportunity loss as a function of the point estimate $\hat{\theta }$ and at its minimum for varying prior distributions.

Substitution of the optimal posterior act obviously fulfills the minimization operation in (29) and integration with respect to the marginal outcome distribution then yields the complete integral term in (29) as:

(35) $$\begin{array}{l} \underset{\left\{0,1,\ldots ,n\right\}}{\int }p_{n}\left(r_{n}\right)\min_{\hat{\theta }\in \left[0,1\right]}\left(\underset{\left[0,1\right]}{\int }p_{n}\left(\theta |r_{n}\right)l_{t}\left(\hat{\theta },\theta \right)d\theta \right)dr_{n}\\ -\min_{\hat{\theta }\in \left[0,1\right]}\int p\left(\theta \right)l_{t}\left(\hat{\theta },\theta \right)d\theta \\ =\frac{\alpha +\beta }{\alpha +\beta +n}\cdot \frac{\alpha \beta }{{\left(\alpha +\beta \right)}^{2}+\left(\alpha +\beta +1\right)} \end{array}$$

For a proof of this identity, please see the Supplementary Material. The quantity (35) may be referred to as the “expected maximal terminal utility,” where the expectation is taken with respect to the possible experimental outcomes. It represents the minimal loss the decision maker has to accept when using the optimal posterior act as point estimate for θ averaged over all possible experimental outcomes. Notably, the expected minimal terminal opportunity loss is monotonically decreasing with sample size and approaches zero for large *n* and is dependent on the choice of the prior distribution. From (35) we see that the optimal sample size for the SSP in the current scenario of a quadratic loss function and a beta prior distribution is a function of the prior distribution (parameters) and the sampling cost constant, which in analogy to (12), we may write as:

(36) $$\begin{array}{rcl} h:{\mathbb{R}}_{\left[0,\infty \right]}\to \mathbb{R},n\mapsto h\left(n\right) & := & \frac{\alpha +\beta }{\alpha +\beta +n}\frac{\alpha \beta }{{\left(\alpha +\beta \right)}^{2}\left(\alpha +\beta +1\right)}\\ +cn. \end{array}$$

Analytical minimization of this function (see Supplementary Material) yields the explicit closed-form solution for the optimal sample size in the current scenario as

(37) $$\begin{array}{l} n^{opt}=\sqrt{\frac{1}{c}\frac{\alpha \beta }{\left(\alpha +\beta \right)\left(\alpha +\beta +1\right)}}-\left(\alpha +\beta \right). \end{array}$$

**Figure 5:**
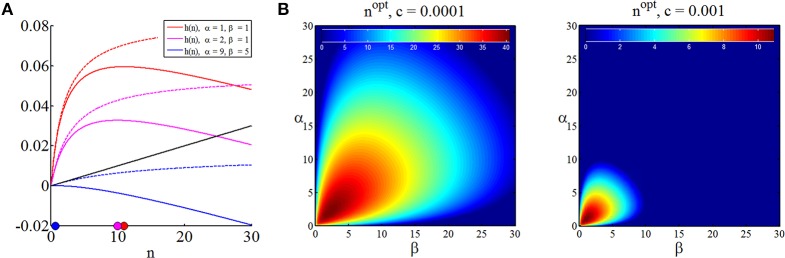
**Optimal sample sizes for parameter point estimation. (A)** For three different prior parameter settings, this panel depicts the expected maximal terminal utility as function of the sample size (dashed lines), the sampling cost for sampling cost constant of *c* = 0.001 (black line) and the resulting functions *h*, and their maxima in the space of sample sizes (colored dots). **(B)** Optimal sample sizes over a range of prior parameter settings α, β ∈ [0, 100] for two sampling cost constants. Higher sampling cost implicates lower optimal sample sizes, while the optimal sample size dependence on the prior parameter size is not affected. In the right panel, sample sizes are optimally zero for high prior certainties about the value of θ.

Note that we have replaced the space of experiments ℕ^0^ by ℝ_[0, ∞]_ in (36) to obtain a differentiable function continuous in *n*, the optimal sample size in discrete terms may then always be found by choosing the closest positive integer to *n*^*opt*^. In Figure 5A, we visualize the expected terminal utility, the function *h*, and its maximum for three different settings of prior parameters. In Figure 5B we visualize the optimal sample size as a function of the prior distribution parameters for two different settings of the sampling cost constant *c*. Notably, the sampling cost constant scales the optimal sample size, but does not affect the functional relationship of optimal sample size and prior distribution [which is, of course, also apparent from (37)].

In summary, assuming, that the decision maker in the SSP (1) is adopting the “inference approach,” (2) is willing to commit to a classical, squared-loss parameter inference scheme, and (3) is willing to quantify its prior uncertainty about the binary payoff distribution parameter using a beta distribution, she may thus read-off the optimal sample size depending on her subjective sampling cost constant *c* > 0 and prior uncertainty captured by the beta distribution parameters α and β about the binary distributions from graphs such as Figure 5B.

## Optimal sample sizes for bayesian parameter inference

Until now we assumed that the decision maker determines the optimal sample size with the aim to obtain a point estimate of the “true state of the world” (that is, the parameter θ of the binary payoff distribution). To this end, the action space *A* of the MEU framework was identified with the state space *S*, both being equal to the interval [0, 1], and the optimal action corresponded to inferring ${\hat{\theta }}_{\alpha ,\beta ,n,r_{n}}^{opt}\in \left[0,1\right]$. Now, we assume that the decision maker is willing to accept a degree of uncertainty about the true state of the world upon selection of the optimal action. To this end, following Bernardo (1997), we assume that the action space corresponds to the space Q of probability distributions over the state space *S*, or more formally:

(38) $$\begin{array}{l} A:=Q:=\left\{q\left(s\right)|q\;is\;a\;probability\;distribution\;on\;S\right\}. \end{array}$$

Note that this space should not be confused with the probability measure over *S* × *Z* as assumed by the MEU framework, but is identified with the action space. The benefit of conceiving the action space as a space of probability distributions over the states is two-fold: First, one may argue that given the limited sample sizes in real-world sampling, the assumption of a point-estimate for the state of the world with its inherent claim to be the single true answer to the inference problem is “overly confident.” Secondly, and perhaps more importantly, it allows for the formulation of the inference approach to the SSP in terms of information theoretic concepts. This has the benefit that the utility functions employed can be derived in a principled manner under the assumption of “quantitative coherence,” and do not assume relatively *ad hoc* forms (e.g., the quadratic loss function considered previously). More specifically, under the assumption of the action space corresponding to the space of probability distributions over states, the fundamental problem in determining an optimal sample size can be framed as a trade-off between maximizing the (well-defined) information about the parameter and minimizing sample cost. In the following, we extend the theoretical framework the Section “The Maximal Expected Utility Framework” by considering a specific type of utility function, and then apply the “Bayesian parameter estimation” variant of the MEU to the SSP. Finally, we employ the same prior distribution as used above and evaluate the resulting optimal sample sizes.

Continuing from the introduction of the terminal utility function *u*_*t*_ in Equation (5), we consider the consequences of identifying the action space with the space Q of probability distributions on the state space. Denoting the members of this space by *q*(*s*), the terminal utility function takes the form:

(39) $$\begin{array}{l} u_{t}\;:Q\times S\to \mathbb{R},\left(q\left(s\right),s\right)\mapsto u_{t}\left(q\left(s\right),s\right). \end{array}$$

As discussed in Bernardo and Smith (1994), a sensible choice for the terminal utility function under the assumption of the action space corresponding to the set of probability distributions over the space of possible states of the world is the “logarithmic score function”:

(40) $$\begin{array}{l} u_{t}^{\ell }\left(q\left(s\right),s\right)=a\log q\left(s\right)+b\left(s\right), \end{array}$$

where *a, b* > 0. In Bernardo and Smith (1994) the use of a logarithmic score function is motivated by the fact that such a function is a natural form of so-called “proper, local score functions” (for a proof, see Bernardo and Smith, 1994, Section 2.7). The ideas behind this are as follows: a “score function” is defined as any smooth function that assigns a real number to each pair of distribution over *s* and value of *s*. A “proper score function” is a score function that takes on its maximal value, if the function *q*(*s*) corresponds to the “posterior” distribution over *s*, that is, in the current scenario the data-conditional distribution *p*_*e*_(*s*|*z*). This condition ensures that the decision maker chooses an action [i.e., a distribution *q*(*s*)] which is coherent with her inference scheme on the space *S* × *Z*. Finally, a score function is said to be “local,” if it depends on the density function *q*(*s*) only through its value at *s*. While the requirements for properness and locality are well-motivated on the background of inferential decision problems for example in scientific contexts (as they, for example, foster honesty), for the application to the SSP, the consequences, rather than the preconditions, of using logarithmic score functions are perhaps more important. Specifically, the use of a logarithmic score function allows for the definition of a number of information-theoretic quantities that formalize the idea that through sampling, the decision maker aims to increase her “knowledge” of “information” about the parameter of interest. Notably, these concepts of “information” are coherent with the general probabilistic theory of information as developed by Shannon (Shannon, 1964; Cover and Thomas, 2006) and prevalent in many modern data analytical frameworks (Bishop, 2007; Barber, 2012; Murphy, 2012), while the original use of “information” quantities, such as the “expected value of perfect information” and the “expected value of sample information” in the classical framework of Raïffa and Schlaifer (1961) was not. In particular, the use of a logarithmic score function permits the definition of “information from data” and the “expected information from an experiment” in an information-theoretic sense. It is this last quantity, which the decision maker can trade-off with the expected cost of sampling in order to come to a judgment of the optimal sample size.

To introduce the notion of “information from data” (Bernardo and Smith, 1994) [or “the amount of information about *s*” (Bernardo, 1997)], we consider again the definition of the optimal sample size with additive utility Equation (14), here adapted for *A*: = Q and the use of a logarithmic score function as defined in (40):

(41) $$\begin{array}{l} n^{opt}=\arg\underset{{}_{n\;\in \;\mathbb{N}}}{\max}(\int_{Z}p_{n}\left(z\right)\underset{q\left(s\right)\;\in \;Q}{\max}\\ \;\left(\int_{S}p_{n}\left(s|z\right)u_{t}^{\ell }\left(q\left(s\right),s\right)dsdz\right)-cn). \end{array}$$

Because the only argument dependent on *q*(*s*) on the right hand side of the above is *u*_*t*_(*q*(*s*), *s*), we may rewrite the above as:

(42) $$\begin{array}{l} n^{opt}=\arg\underset{{}_{n\;\in \;\mathbb{N}}}{\max}(\int_{Z}p_{n}\left(z\right)\\ \;\int_{S}p_{n}\left(s|z\right)\underset{{}_{q\left(s\right)\in Q}}{\max}\left(u_{t}^{\ell }\left(q\left(s\right),s\right)\right)dsdz-cn). \end{array}$$

By definition, the logarithmic score function is maximized for the posterior distribution *p*_*e*_(*s*|*z*) and we obtain:

(43) $$\begin{array}{l} n^{opt}=\arg\max_{n\in \mathbb{N}}\left(\underset{Z}{\int }p_{n}\left(z\right)\underset{S}{\int }p_{n}\left(s|z\right)u_{t}^{\ell }\left(p_{n}\left(s|z\right),s\right)ds\;dz-cn\right). \end{array}$$

If one additionally defines the coefficient *a* to be 1 and, and in analogy to the notion of terminal opportunity loss above, the terminal utility of reporting the prior distribution over states *p*(*s*) to be zero (Bernardo, 1997) one has:

(44) $$\begin{array}{l} u_{t}\left(p\left(s\right),s\right)=0\Leftrightarrow b\left(s\right)=-\log p\left(s\right). \end{array}$$

Then, because

(45) $$\begin{array}{l} u_{t}^{\ell }\left(p_{n}\left(s|z\right),s\right)=\log p_{n}\left(s|z\right)-\log p\left(s\right), \end{array}$$

the inner integral in (43) can be written as:

(46) $$\begin{array}{l} KL\left(p_{n}\left(s|z\right)|\;p\left(s\right)\right):=\underset{S}{\int }p_{n}\left(s|z\right)\log\left(\frac{p_{n}\left(s|z\right)}{p\left(s\right)}\right)ds. \end{array}$$

The quantity (46) is the well-known Kullback-Leibler divergence (Kullback and Leibler, 1951) between the posterior distribution *p*_*n*_(*s*|*z*) and the prior distribution *p*_*n*_(*s*). The Kullback-Leibler divergence is a versatile quantity with ubiquitous applications in probabilistic modeling and here appears in form of a terminal utility as a consequence of the MEU framework with logarithmic score functions. In this context, and if evaluated in a data dependent manner between a posterior and prior distribution, it is referred to as “information from data.” In sum, we have reformulated (41) as

(47) $$\begin{array}{l} n^{opt}=\arg\max_{n\in \mathbb{N}}\left(\underset{Z}{\int }p_{n}\left(z\right)KL\left(p_{n}\left(s|z\right)|p\left(s\right)\right)dz-cn\right). \end{array}$$

The remaining integral term (the expected KL divergence between the posterior and prior distribution over states of the world under the marginal distribution of experimental outcomes) is referred to as the “expected information from data,” which we will denote here by the function:

(48) $$\begin{array}{l} I:{\mathbb{N}}^{0}\to \mathbb{R},n\mapsto I\left(n\right)=\int p_{n}\left(z\right)\int p_{n}\left(s|z\right)\log\left(\frac{p_{n}\left(s|z\right)}{p\left(s\right)}\right)dsdz. \end{array}$$

As noted by Lindley (1956) this quantity may alternatively be expressed as the (experiment-dependent) mutual information between the random variable representing possible states of the world *s* and the random variable representing experimental outcomes *z*, i.e.,

(49) $$\begin{array}{l} I\left(n\right)=\iint p_{n}\left(s,z\right)\log\left(\frac{p_{n}\left(s,z\right)}{p_{n}\left(z\right)p\left(s\right)}\right)dsdz. \end{array}$$

Equation (49) captures the intuition that the task of selecting a good sample size (or, more generally, designing a good experiment) corresponds to maximizing the information that potential outcomes contain about the underlying state of the world [which, for real experiments has to be traded off against the cost of experimentation as apparent from (47)]. We next apply these general results to the SSP.

### Optimal sample sizes for logarithmic scoring functions and beta prior

As in the Section “Optimal Sample Sizes for Parameter Point Inference,” we define the set of possible states of the worlds as the true, but unknown value of the Bernoulli distribution parameter by *S*: = [0, 1]. Equally, we identify *E*: = ℕ_0_ and assume Z to be the sufficient outcome space ℕ_*n*_ of a binomial sampling approach. As discussed above, we assume *A* to be the space of probability distributions on [0, 1], here represented by probability density functions:

(50) $$\begin{array}{l} Q:=\left\{q\left(\theta \right)\;q\left(\theta \right)>0,\overset{1}{\underset{0}{\int }}q\left(\theta \right)d\theta =1,\theta \in \left[0,1\right]\right\}. \end{array}$$

Note that the assumption of the elements of *Q* being probability density functions is a mere notational convenience. The results could equally well be formulated in terms of probability mass functions defined on suitably chosen partitions of [0, 1] (see Bernardo and Smith, 1994). In summary, we thus assume the MEU problem space:

(51) $$\begin{array}{l} A\times S\times E\times Z:=Q\times \left[0,1\right]\times {\mathbb{N}}^{0}\times {\mathbb{N}}_{n}^{0}. \end{array}$$

The form of the utility function was discussed in detail above. Further, as above we assume the following probability measure on the Cartesian product of the space of states of the world and experimental outcomes:

(52) $$\begin{array}{l} p_{n}\left(\theta ,r_{n}\right)=p\left(\theta \right)p_{n}\left(r_{n}|\theta \right)=Be\left(\theta ;\alpha ,\beta \right)Bi\left(r_{n};n,\theta \right), \end{array}$$

again implicating the data conditional distribution and marginal distributions:

(53) $$\begin{array}{lll} p_{n}\left(\theta |r_{n}\right) & = & Be\left(\theta ;\alpha +r_{n},\beta +n-r_{n}\right)\text{ and}\\ p_{n}\left(r_{n}\right) & = & Bb\left(r_{n};\alpha ,\beta ,n\right). \end{array}$$

To obtain the optimal sample size, we now consider Equation (47), which, based on the specifications above, evaluates to:

(54) $$\begin{array}{lll} n^{opt} & = & \arg\max_{n\in \mathbb{N}}(\underset{{\mathbb{N}}_{n}^{0}}{\int }p_{n}\left(r_{n}\right)KL(Be(\theta ;\alpha \\ +r_{n},\beta +n-r_{n})|Be\left(\theta ;\alpha ,\beta \right))dr_{n}-cn). \end{array}$$

In this equation, the integral term mirrors the minimized posterior terminal opportunity loss of Equation (29) and intuitively corresponds to the expected KL-divergence between the posterior and prior beta distributions under the marginal distribution of the data. The KL-divergence between two beta distributions is well-known (Liu et al., 2006) and the function *h* specified in (12) can be expressed as:

(55) $$\begin{array}{l} h:{\mathbb{N}}^{0}\to \mathbb{R},n\mapsto h\left(n\right)\\ :\text{​}=\sum_{r_{n}\;=\;0}^{n}((\frac{\Gamma \left(\alpha +\beta \right)}{\Gamma \left(\alpha \right)\Gamma \left(\beta \right)\Gamma \left(\alpha +\beta +n\right)}\left(\begin{array}{c} n\\ r_{n} \end{array}\right)\\ \Gamma \left(\alpha +r_{n}\right)\Gamma \left(\beta +n-r_{n}\right))(\ln\left(\frac{\Gamma \left(\alpha +\beta +n\right)}{\Gamma \left(\alpha +r_{n}\right)\Gamma \left(\beta +n-r_{n}\right)}\right)\\ -\ln\left(\frac{\Gamma \left(\alpha +\beta \right)}{\Gamma \left(\alpha \right)\Gamma \left(\beta \right)}\right)+r_{n}\psi \left(\alpha +r_{n}\right)+\left(n-r_{n}\right)\psi \left(\alpha +r_{n}\right)\\ +n\psi \left(\alpha +\beta +n\right)))-cn. \end{array}$$

**Figure 6:**
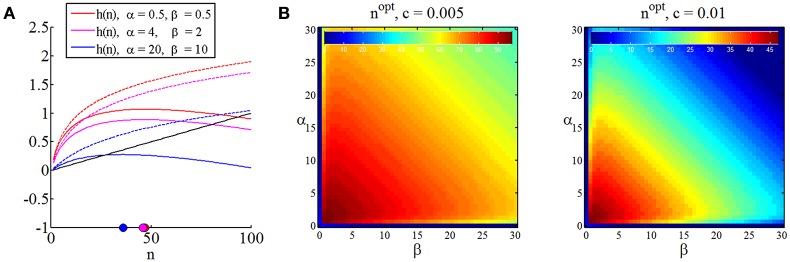
**Optimal sample size for Bayesian parameter inference**. **(A)** For three settings of the prior distribution parameters, this panel depicts the expected information from data (dashed lines), the sampling utility (negative sampling cost) for a cost constant of *c* = 0.02 (black line), the function *h* as defined in Equation (55), and its numerically determined maximum. **(B)** For two cost constants, the panels depict the optimal sample sizes as a function of the prior parameters. As for the optimal sample sizes in the case of parameter point inference, the optimal sample size is symmetric in α and β, the optimal sample size is inversely proportional to the sampling cost, and sampling sizes of zero are optimal for high prior certainties.

For a proof, please refer to the Supplementary Material. Unfortunately, unlike the corresponding function in the case of parameter point inference, the function *h* in (55) is not readily maximized analytically. However, because the function shows strictly concave behavior, its maximum may be identified by numerically evaluating its values for increasing *n* until *h*(*n* + 1) ≤ *h*(*n*). Mirroring Figure 5 for the case of parameter point inference, in Figure 6 we depict the minimized posterior terminal utility of Equation (55), the function *h* of Equation (55), and optimal sample sizes under the assumption of Bayesian parameter inference for different values of the sampling cost constant *c*.

In summary, assuming that the decision maker in the SSP (1) is adopting the “inference approach,” (2) is willing to commit to a Bayesian parameter estimation scheme in which the utility of sampling is expressed as the information (in an information-theoretic sense) about the binary payoff distribution parameter, and (3) is willing to quantify her prior uncertainty about the distribution parameter using a beta distribution, she may thus read-off the optimal sample size depending on its subjective sampling cost constant *c* > 0 and prior uncertainty captured by the beta distribution parameters α and β about the binary distributions from graphs such as Figure 6B. Compared to the approach in Section Optimal Sample Sizes for Parameter Point Inference, the decision maker here has the additional freedom of probabilistically selecting a parameter value, for example, by choosing the mode of the posterior distribution (maximum-a-posterior estimator), or such that it falls into a 95%-credibility interval.

## Numerical solutions

In our application of the MEU framework for parameter point or interval probability estimation we have so far assumed analytically tractable parameter prior probability distributions and terminal utility functions mostly for mathematical convenience. However, the MEU framework is by no means limited to these special classes of probability distributions and terminal utility functions. In this Section we demonstrate how the optimal sample size for the inference approach to the SSP can be derived with the help of a computer for arbitrary, but numerically evaluable, prior distributions and terminal utility function. As we elaborate below, this approach is of particular relevance for applications of the theory developed here in an experimental context. Note that our demonstration merely serves as a proof-of-principle and does not aim for the systematic evaluation of the errors introduced by the numerical approximation of analytic quantities or attempts to provide an in any way exhaustive coverage of possible prior and terminal utility functions.

**Figure 7:**
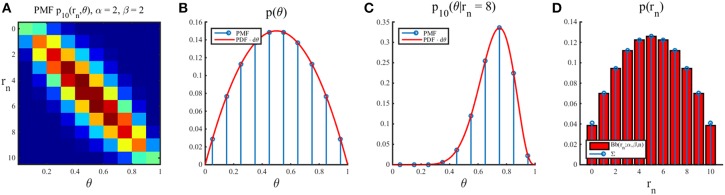
**Numerical Bayesian inference for a Binomial likelihood function**. For a discretization of the state space into 10 equally spaced bins centered at 0.05, 0.15,…, 0.95, beta distribution prior parameters of α = β = 2, and a sample size of *n* = 10, the panels depict, from left to right **(A)** the joint distribution over states θ and outcomes *r*_*n*_, **(B)** the probability mass function (blue) resulting from an approximation of the corresponding probability density function (red), **(C)** the posterior distribution for the outcome observation *r*_*n*_ = 8 in both analytically evaluated probability density form (red) and numerically evaluated probability mass function form (blue), and **(D)** the discrete marginal outcome distribution in form of the analytically evaluated (red) and numerically evaluated probability mass function (blue). Note the difference in scale between the first and second panel.

With respect to the MEU framework, we first note that if one specifies the marginal distribution *p*(θ) not by means of a probability density function as above, but by means of a probability mass function for an appropriately chosen discretization of the parameter space, the data-conditional parameter distribution *p*_*n*_(θ|*r*_*n*_) also assumes the form of a probability mass function, which can be evaluated according to Bayes theorem as follows:

(56) $$\begin{array}{rcl} p_{n}\left(\theta =\theta_{i}|r_{n}\right) & = & \frac{p_{n}\left(r_{n}|\theta =\theta_{i}\right)p\left(\theta =\theta_{i}\right)}{\underset{\theta_{i}\in \Theta }{\sum }p\left(r_{n},\theta =\theta_{i}\right)}\\ = & \frac{Bi\left(r_{n};n,\theta =\theta_{i}\right)p\left(\theta =\theta_{i}\right)}{\underset{\theta_{i}\in \Theta }{\sum }Bi\left(r_{n};n,\theta =\theta_{i}\right)p\left(\theta =\theta_{i}\right)}\\ \text{for }i=1,\ldots ,d, \end{array}$$

where *d* ∈ ℕ denotes the (finite) number of chosen parameter values of interest. This approach is visualized in Figure 7.

**Figure 8:**
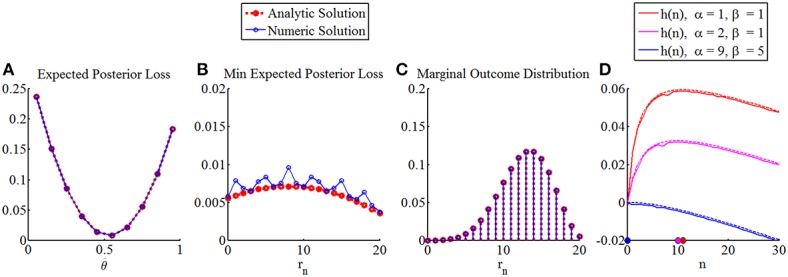
**Numerical replication of optimal sample sizes for parameter point estimation with squared error loss function and beta prior distribution**. From left to right, the panels depict: **(A)** the expected posterior loss in analytical (red) and numerical (blue) form, for prior parameters α = 9, β = 5 a sample size of *n* = 20, and an observed outcome of *r*_*n*_ = 9, **(B)** for the same prior parameters and sample size the minimal expected posterior loss (notably, the fact that in the analytical treatment the sample size is conceived for a continuous sample size variable (in the current visualization of course approximated by a discretization of the sample size space with bin sizes smaller than 1), introduces the largest errors that result from an numerical approach), **(C)** the marginal distribution over outcomes in both numerical and analytical form, and finally **(D)** the function *h*(*n*) for the same sampling cost constants and prior parameter settings as in Section The Maximal Expected Utility Framework in both numerical form (straight lines) and analytical form (dashed lines).

We further note that the integration operations can, for finite discrete state and outcome spaces and probability mass functions defined over these spaces, be evaluated by means of scalar products. Finally, the respective maximization operations can be evaluated using standard list sorting techniques available in numerical computing. Figure 8 depicts the numerical replication of the analytical results obtained in the Section “Optimal Sample Sizes for Parameter Point Inference” for the case of a beta prior distribution and a squared error loss function. Here, some numerical error is introduced by the discretization of state space. However, the larger errors are introduced by the treatment of the sample size as a continuous variable in the analytical case.

**Figure 9:**
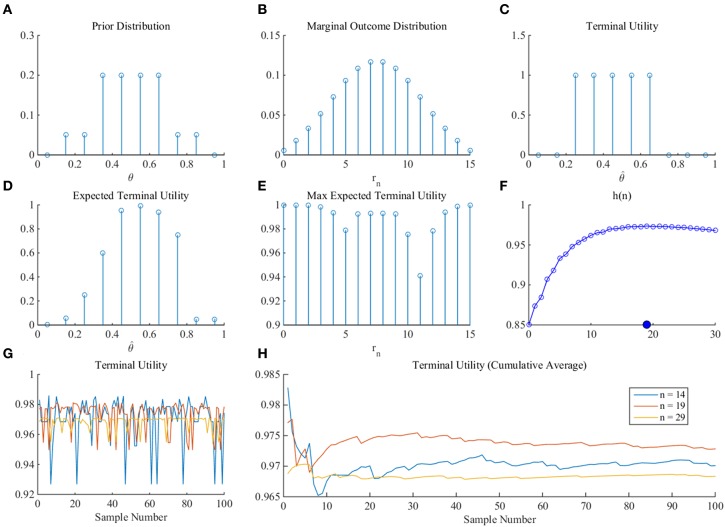
**Numerical proof-of-principle**. For a detailed discussion of this figure, please refer to the main text.

As a proof-of-principle that the MEU framework can yield an optimal sample size for arbitrary prior distributions and terminal utility function, we consider prior probability mass and terminal utility functions for a discretization of the state space into 10 equally spaced bins (Figure 9). Specifically, we specify the following prior distribution over states (Figure 9A).

(57) $$\begin{array}{rcl} p\left(\theta =0.05\right) & = & p\left(0.95\right)=0\\ p\left(\theta =0.15\right) & = & p\left(\theta =0.25\right)=p\left(\theta =0.75\right)\\ = & p\left(\theta =0.85\right)=0.05\\ p\left(\theta =0.35\right) & = & p\left(\theta =0.45\right)=p\left(\theta =0.55\right)\\ = & p\left(\theta =0.65\right)=0.2. \end{array}$$

As previously, we use the binomial distribution for *p*_*n*_(*r*_*n*_|θ) and numerically derive the marginal distribution over outcomes (Figure 9B). For the terminal utility function, we define an “inflated 1-0 utility function” by setting:

(58) $$\begin{array}{rcl} u_{t}:\left[0,1\right]\times \left[0,1\right] & \to & \left\{0,1\right\},\left(\hat{\theta },\theta \right)\mapsto u_{t}\left(\hat{\theta },\theta \right)\\ :=\{\\ 1,\;|\hat{\theta }-\theta |\leq 0.2\\ 0,\;|\hat{\theta }-\theta |>0.2, \end{array}$$

which is shown for θ = 0.45 in Figure 9C. Based on the numerical scheme introduced above, we then evaluate the necessary functions and quantities (Figures 9D–F) and arrive, for a sampling cost constant of *c* = 0.001, at an optimal sample size of *n*^*opt*^ = 19.

In summary, assuming that the decision maker (1) adopts the inference approach, (2) commits to arbitrary, but numerically evaluable prior distribution probability mass and utility functions, and (3) has the numerical computing facilities, she may thus read-off the optimal sample size depending on her subjective sampling cost constant *c*>0 and prior uncertainty from graphs such as Figure 9F.

Leaving technicalities aside, we next elaborate on the applicability of the numerical solutions discussed above in a concrete experimental context. We address this scenario first from the perspective of the decision maker, i.e., the experimental participant, and then from the perspective of the experimenter.

Consider an experimental participant faced with the SSP. In line with the inferential notion of our framework, we assume that the participant would like to solve the question of how many samples to draw before deciding whether to take a final draw with economic consequences by means of estimating the expected value of the SSP. As we focused our discussion on estimating the binary payoff distribution parameter θ, we have to implicitly assume that the participant is aware of the SSP's binary payoff distribution functional form and has knowledge about the values of the possible outcomes (e.g., by having been exposed to experimental trials of the same task previously). Our framework next assumes that the participant has a means to quantify her initial uncertainty about the value of θ in terms of a prior distribution over discrete possible values of θ. For the current purposes, we assume that this has resulted in the distribution shown in Figure 9A. By specifying this marginal distribution over θ and under the assumption of knowledge of the binary payoff character of the problem, the experimental participant has now implicitly specified a marginal distribution over experimental outcomes *r*_*n*_ (number of observations of *x*_1_) for each sample size *n* considered. For the prior distribution of Figure 9A, this marginal distribution of experimental outcomes is shown in Figure 9B. This specific distribution corresponds to the subjective belief of the probability of observing the outcomes of 0, 1, …, 15 occurrences of *x*_1_ in a pre-specified sample size of *n* = 15. We next assume that the participant can quantify her terminal utility of misestimating the parameter θ. Like the initial uncertainty over the value of θ, this terminal utility may take many forms. Here, we assume that the participant would like to avoid over- or under-estimating θ by 0.2, which we can formalize as the inflated 1-0 loss function noted in (58) and visualized for an assumed true, but unknown, value of θ^*^ = 0.45 in Figure 9C. Note that in the current framework this loss function has only hypothetical, subjective applications. In other words, at no point is the true, but unknown, value of θ actually revealed to the participant, such that the participant could evaluate the degree of her over- or under-estimation in practice. Finally, our framework implies that the participant can trade-off her terminal utility of correctly estimating the parameter θ with the cost (“negative utility”) *c* of obtaining a single observation. Note that this sampling cost can be conceived as the (intrinsic or extrinsic) time-constraint of the participant for carrying out the sampling—if more samples are drawn, the experimental procedure will take longer, and the participant may not want to sit in the experiment for the rest of her life. Having now specified all essential components, our framework implies that the experimental decision maker would like to determine the best sample size that allows her to maximize her subjective utility (terminal utility and negative sampling cost). We may simply frame this as that the participant would like to “behave optimally” with respect to her initial uncertainty over θ and her economic preferences. Importantly, this concept of optimal behavior is completely subjective—from an objective viewpoint, there is no guarantee that, e.g., the initial uncertainty over θ is not completely misled with respect to the true, but unknown, value of θ. In other words, our framework does not address an objective or absolute form of optimality, but rather a subjective or relative form. Given all of the above, the participant can now evaluate her optimal sample size by firstly integrating her terminal utility function over the outcome specific posterior distribution (expected terminal utility Figure 9D), maximizing this function with respect to an estimate $\hat{\theta }$ (maximized expected terminal utility Figure 9E), integrating the latter with respect to the marginal distribution over outcomes, and finally maximizing the resulting sample size-dependent function with respect to the sample size while accounting for sample costs (Figure 9F). From an “observed frequency” perspective, as shown in Figure 9G (sampled terminal utilities) and Figure 9H (cumulative averages of the sampled terminal utilities), this optimal sample size of *n* = 19 maximizes the expected utility over samples from the subjective marginal distribution of experimental outcomes, as compared to under- and over-sampling (*n* = 14 or *n* = 29, respectively). Note, however, that this observed-frequency perspective, while illustrative, is not coherent with the interpretation of the participant's probabilistic model as a quantification of uncertainty, and that under this interpretation, one would content with the analytically determined expected value.

We next consider the experimental applicability of our numerical framework from the perspective of the experimenter. By considering the framework discussed here, we obviously assume that the experimenter is led by the intuition that the participant's prior assumptions about the state of the world and economic preferences are of importance when studying decision making under uncertainty. More specifically, an experimenter may view the framework discussed herein from two perspectives. Firstly, the experimenter may conceive the proposed framework as a normative “null” model, which has no psychological plausibility, but can serve as an objective predictor for subjectively optimal behavior. In other words, assuming that the experimenter has made the participant's prior assumptions, terminal utility function, and sampling cost, explicit (for example by having explained the binary payoff distribution character of the SSP to the participant, having the participant revealed her prior belief over discrete values of θ, for example by means of a visual analog scale, and likewise having revealed the participant's terminal utility function and sampling cost), the experimenter can test whether the participant behaves in accordance with her subjective preferences or not. In case of the former, the question arises, how the participant's neurocognitive apparatus is able to implement (or at least approximate) the non-trivial computations involved. In case of the latter, the question arises, which cognitive processes may distort the mapping from prior beliefs and preferences to selected sample sizes—which in turn may lead to more psychological plausible accounts of the decision processes in the SSP. Secondly, the experimenter may conceive the framework as a valid working hypothesis and, by fixing or inducing specific components of the framework, study others. For example, assume that the participant has specified her prior beliefs over the values of θ and her sampling cost constant *c*. Based on her actual experimentally observed sample sizes (and, of course, under the other assumptions of the framework, such as the 1-0 loss function), the experimenter can now determine the degree of over- and underestimation of θ the participant allows herself. Complementary, assume that the participant has revealed her degree of preferred misestimation of θ and sampling cost constant *c* to the experimenter. Then, based on the observed sampling behavior, the experimenter can obtain an approximation of the prior beliefs over the values θ. Finally, by revealing the prior distribution, misestimation preference, and observed sample size, the experimenter can study the cost that the participant assigns to a single draw. Additional possibilities for using the current framework in the second way arise by experimentally inducing prior assumptions, terminal utility functions, and sampling costs and then observing the behavioral consequences in sampling behavior. We further discuss the experimental value of the framework in the Discussion.

## Discussion

In this study we have shown how a normative benchmark for optimal sample sizes in the DFE sampling paradigm can be developed based on results from classical statistical decision theory. More specifically, we have shown that assuming an inference approach to the sampling problem in DFE, optimal sample sizes are dependent on the desired inference type and can be quantitatively related to the decision maker's prior beliefs about the problem, the decision maker's value assigned to identifying the correct solution, and the decision maker's cost assigned to each sample. We conclude with discussing the benefits and limitations of this framework for generating testable predictions and point to potential applications of the framework in experimental cognitive psychology.

Perhaps the most fundamental benefit of the MEU framework in the context of DFE is that it is explicit and constructive: upon specification of the necessary concepts (the state, action, experimental, and outcome spaces, the utility function, and the probability measure on the product of experimental and outcome space) it will yield an optimal sample size. From the perspective of behavioral experimentation this is helpful, because search behavior in DFE can be tested against quantitative predictions. Further, because of its generality, the MEU framework can be adapted to a wide range of conceivable utility functions (for example those incorporating a notion of risk-sensitivity Shen et al., 2014), probabilistic assumptions beyond binary payoff distributions (for example sampling from Gaussian distributions, Daunizeau et al., 2010), and prior distributions. Finally, because it is explicit with respect to its concepts and assumptions, it can serve as a reference point for more psychological plausible accounts of information search in DFE. For example, it may be argued that the assumption of a prior distribution over states and its ensuing observation-based update to a posterior distribution is a cognitively impossible task. If the aim is to find a framework that does not require the specification of a joint probability measure on world states and experimental outcomes, but is still constructive insofar as it allows for the derivation of quantitative sample size predictions, then the MEU framework may serve as a starting point from which assumptions can successively be removed.

Perhaps the most fundamental limitation of employing the MEU framework as a generative model for behavioral DFE data is that it is, as presented here, “non-identifiable.” By this we mean, as shown by Figures 5B, 6B, 9F, that a given optimal sample size can be explained by a wide range of combinations of prior distributions and utility functions. This issue is technically related, but not identical to, the “complete class theorem.” However, this weakness may also be a strength, because it guides the experimenter directly to interesting and testable hypotheses and well-controlled experiments: by experimentally controlling a pre-defined subset of variables (for example, the participant's prior beliefs and sampling cost), observed sample sizes can be used to infer the possible utility (value) functions on which basis a person operates. Analogous arguments can be made in order to infer participant's prior beliefs or subjective sampling costs. Note however, that as it stands the MEU framework for DFE cannot serve as an inferential (or “generative”) model of empirical data, because it has not been embedded into a probabilistic framework that allows for model parameter estimation and comparison by means of evaluation of marginal data likelihoods. However, this is a technical issue, and its solution readily conceivable, even if not readily carried out. As an example, Daunizeau et al. (2010) have recently demonstrated the formal probabilistic embedding of similar behavioral data models by capitalizing on variational Bayesian frameworks, while a popular strategy in mathematical psychology is the embedding in MCMC schemes in combination with *ad hoc* model selection criteria (Lee and Wagenmakers, 2014). Combining the current framework with suitable identifiability constraints and one of the mentioned scientific inference approaches thus allows for overcoming this limitation.

Because of its indefiniteness, an unlimited set of objections can be raised against the current approach from the perspective of cognitive process modeling. We thus limit ourselves to a set of objections for which we see constructive rejoinders at present. A first objection may be that the current application of the MEU framework assumes that participants have knowledge of the (to be) observed outcomes such that estimating the state of the world, i.e., the parameter θ ∈ [0, 1] in the SSP is actually the only necessary action. We agree that this assumption has been made here (for experimental approaches in DFE that work on a similar basis, see selected experiments in Erev et al., 2008; Rakow and Miler, 2009). A solution to this objection may be to generalize the inference approach to the estimation of the tuple (*x*_1_, *x*_2_, θ) of observed outcomes and parameter, where the sufficient statistics for the outcomes may correspond to the first observations. It should also be noted that in general, upon sampling, both outcomes will have been observed, permitting for the evaluation of the expected value estimate based on the inferred probability parameter values. A second objection is that it is implausible that participants in DFE studies evaluate optimal sample sizes for each payoff distribution prior to starting the sampling. Instead, they may after each observation (or sets thereof) decide whether to (a) terminate the exploration phase and continue to the final incentivized draw, (b) continue sampling from the currently investigated payoff distribution, or (c) terminate sampling from the currently investigated payoff distribution and to start (or continue) to sample from another payoff distribution. A model class appropriate to capture these intuitions is offered by the theory of (partially observable) Markov decision processes (Wiering and Otterlo, 2012). A valuable future contribution would be the explicit comparison of the numerical sample size predictions offered by the MEU and POMDP frameworks under identical prior, utility, and sampling cost assumptions.

Finally, we note that (Vul et al., 2014) have recently addressed the question of optimal decisions based on sampling and related their work to the DFE literature. However, as Vul et al. (2014) point out, their considerations address a different (postulated) phenomenon to that commonly considered in DFE studies and the current manuscript: Vul et al. (2014) address—inspired by sampling-based approximate Bayesian inference techniques such as Gibbs sampling—the postulated cognitive “internal” sampling of the decision maker's posterior distribution over states of the world *p*_*e*_(*s*|*z*). They conclude that a few samples from the posterior distribution can suffice for an agent to make “optimal” decisions in a given choice ecology. At present, because of the fundamental difference of which distribution is being sampled, their approach is not easily related to the MEU framework and such an analytical treatment is beyond the scope of the current manuscript. However, their approach is of high relevance for the formulation of alternative generative models for experimental DFE inference by focusing on “optimal decisions” rather than “optimal sample sizes,” as we do in the current manuscript. Future analytical studies may shed light on the precise relationship between the MEU framework considered here and the work by Vul et al. (2014).

In summary, a broad empirical literature on DFE has developed over the last decade in behavioral psychology, which has shown that human choice behavior can remarkably differ depending on how information is presented and sampled for uncertain choices with economic consequences. However, so far few attempts have been made to study the quantitative nature of human sampling behavior in DFE by means of computational modeling. Specifically, no normative benchmark has been developed that would allow to judge whether and when the observed sample sizes drawn by human observers are “reasonable.” In this study, we related the DFE sampling paradigm to the classical and modern literature on statistical decision making and reviewed and extended a framework based on which such a normative benchmark can be developed. Specifically, we have shown how, under a probabilistic inference assumption, the optimal sample size in DFE can be quantitatively related to the decision maker's preferred type of inference, prior beliefs about the payoff distributions at hand, and utility assigned to the inference's precision. Because of its quantitative nature, the framework introduced here has yielded directly testable predictions for the behavioral study of DFE. Moreover, given the strong conceptual similarity between the DFE sampling paradigm and evidence accumulation schemes as prevalent in research on perceptual decision making, we believe that the current study addresses key theoretical aspects of decision making under dynamic subjective uncertainty. Finally, we believe that the current study lays an important foundation for future theoretical efforts on the computational description of human behavior in the DFE sampling paradigm and provides a useful basis towards their experimental validation.

### Conflict of interest statement

The authors declare that the research was conducted in the absence of any commercial or financial relationships that could be construed as a potential conflict of interest.
