# Supplementary material for: A normative inference approach for optimal sample sizes in decisions from experience
Source: Front Psychol. 2015 Sep 10;6:1342. doi: 10.3389/fpsyg.2015.01342 (PMC4564691; doi:10.3389/fpsyg.2015.01342)
Supplement: Supplementary file 1 [file DataSheet1.PDF]

Supplementary Material of Ostwald D, Starke L and Hertwig R (2015) A normative inference approach for optimal sample sizes in decisions from experience. Front. Psychol. 6:1342.

Proof of (33) and (34)

We have

$$\int_{[0,1]} l_t(\hat{\theta},\theta) p_n(\theta|r_n) d\theta := \langle l_t(\hat{\theta},\theta)\rangle_{p_n(\theta|r_n)} \tag{33.1}$$

$$= \langle (\theta - \hat{\theta})^2 \rangle_{p_n(\theta|r_n)}$$

$$= \langle \theta^2 - 2\theta\hat{\theta} + \hat{\theta}^2 \rangle_{p_n(\theta|r_n)}$$

$$= \langle \theta^2 \rangle_{p_n(\theta|r_n)} - 2\langle \theta \rangle_{p_n(\theta|r_n)} \hat{\theta} + \hat{\theta}^2$$

$$= V_{p_n(\theta|r_n)}(\theta) + \left(E_{p_n(\theta|r_n)}(\theta)\right)^2 - 2\langle \theta \rangle_{p_n(\theta|r_n)} \hat{\theta} + \hat{\theta}^2$$

$$= V_{p_n(\theta|r_n)}(\theta) + \left(E_{p_n(\theta|r_n)}(\theta) - \hat{\theta}\right)^2$$

The above may now be evaluated using the well-known expectation and the variance of a beta distribution $Be(\theta;\alpha,\beta)$ (see e.g., (Bernardo & Smith, 1994), Section 3.2) in parameterized form by

$$E_{Be(\theta;\alpha,\beta)} = \frac{\alpha}{\alpha+\beta} \text{ and } V_{Be(\theta;\alpha,\beta)} = \frac{\alpha\beta}{(\alpha+\beta)^2(\alpha+\beta+1)} \tag{33.2}$$

We thus have

$$\int_{[0,1]} l_t(\hat{\theta},\theta) p_n(\theta|r_n) d\theta = \frac{(\alpha+r_n)(\beta+n-r_n)}{(\alpha+\beta+n)^2(\alpha+\beta+n+1)} + \left(\frac{\alpha+r_n}{\alpha+\beta+n} - \hat{\theta}\right)^2 \tag{33.3}$$

Because $\alpha, \beta > 0$ and $n \geq r_n$ and because only the latter term in the expression above is a function of $\hat{\theta}$, we have

$$\arg\max_{\hat{\theta}\in[0,1]} \int_{[0,1]} p_n(\theta|r_n)\left(-l_t(\hat{\theta},\theta)\right) d\theta = \arg\min_{\hat{\theta}\in[0,1]} \left(\frac{\alpha+r_n}{\alpha+\beta+n} - \hat{\theta}\right)^2 \tag{33.4}$$

The optimal point estimate (or act) for a given set of prior parameters α, β, sample size $n \in \mathbb{N}^0$ and observed outcome $r_n \in \mathbb{N}_n^0$ α, β (with $r_0 := 0$) is thus

$$\theta_{\alpha,\beta,n,r_n}^{opt} := \frac{\alpha+r_n}{\alpha+\beta+n} \tag{33.5}$$

Notably, from (33.1) it follows that

$$\int_{[0,1]} l_t\left(\theta^{opt}_{\alpha,\beta,n,r_n}, \theta\right) p_n(\theta|r_n) d\theta = V_{p_n(\theta|r_n)}(\theta) \tag{33.6}$$

and, when considering integration with respect to the marginal distribution $p_n(\theta)$ that

$$\min_{\hat{\theta}\in[0,1]]} \int p(\theta)\, l_t(\hat{\theta}, \theta) d\theta = \int p(\theta) l_t\left(\theta^{opt}_{\alpha,\beta,n,r_n}, \theta\right) d\theta = V_{p_n(\theta)}(\theta) \tag{33.7}$$

Proof of (35)

Above, we have shown that

$$\int_{[0,1]} l_t\left(\theta^{opt}_{\alpha,\beta,n,r_n}, \theta\right) p_n(\theta|r_n) d\theta = V_{p_n(\theta|r_n)}(\theta) = \frac{(\alpha+r_n)(\beta+n-r_n)}{(\alpha+\beta+n)^2(\alpha+\beta+n+1)} \tag{35.1}$$

and

$$\max_{\hat{\theta}\in[0,1]]} \int p(\theta) \left(-l_t(\hat{\theta}, \theta)\right) d\theta = V_{p_n(\theta)}(\theta) \tag{35.2}$$

We may thus write

$$\int_{\{0,1,\ldots,n\}} \max_{\hat{\theta}\in[0,1]} \left\{\int_{[0,1]} p_n(\theta|r_n) \left(-l_t(\hat{\theta}, \theta)\right) d\theta\right\} p_n(r_n)\, dr_n + \max_{\hat{\theta}\in[0,1]]} \left\{ \int p(\theta) \left(-l_t(\hat{\theta}, \theta)\right) d\theta\right\}$$

$$= V_{p_n(\theta)}(\theta) - E_{p(r_n)}\left(V_{p_n(\theta|r_n)}(\theta)\right) \tag{35.3}$$

or in other words: the expected minimal posterior opportunity loss corresponds to the expectation of the posterior variance of with respect to the marginal distribution of. Following the approach in (Pratt et al., 1995), this quantity may be economically evaluated by firstly noting that

$$V_{p(\theta)}(\theta) = E_{p(r_n)}\left(V_{p_n(\theta|r_n)}(\theta)\right) + V_{p(r_n)}\left(E_{p_n(\theta|r_n)}(\theta)\right) \tag{35.4}$$

and thus

$$E_{p(r_n)}\left(V_{p_n(\theta|r_n)}(\theta)\right) = V_{p(\theta)}(\theta) - V_{p(r_n)}\left(E_{p_n(\theta|r_n)}(\theta)\right) \tag{35.5}$$

To write (35.4) in terms of the prior distribution parameters and the sample size, we secondly note that the variance of the posterior expectation of θ with respect to $p(r_n)$ can be written as

$$V_{p(r_n)}\left(E_{p_n(\theta|r_n)}(\theta)\right) = \frac{n}{\alpha+\beta+n} \cdot V_{p(\theta)}(\theta) \tag{35.6}$$

We thus have

$$\begin{aligned} E_{p(r_n)}\left(V_{p_n(\theta|r_n)}(\theta)\right) &= \frac{\alpha+\beta+n}{\alpha+\beta+n} \cdot V_{p(\theta)}(\theta) - \frac{n}{\alpha+\beta+n} \cdot V_{p(\theta)}(\theta) \\ &= \frac{\alpha+\beta}{\alpha+\beta+n} \cdot V_{p(\theta)}(\theta) \\ &= \frac{\alpha+\beta}{\alpha+\beta+n} \cdot \frac{\alpha\beta}{(\alpha+\beta)^2+(\alpha+\beta+1)} \end{aligned} \tag{35.7}$$

Proof of (37)

We consider minimization of the differentiable function

$$f: \mathbb{R}_{[0,\infty]} \to \mathbb{R}, n \mapsto f(n) \coloneqq \frac{\alpha+\beta}{\alpha+\beta+n} \frac{\alpha\beta}{(\alpha+\beta)^2+(\alpha+\beta+1)} + cn \tag{37.1}$$

in lieu of the function of interest proper. However, because the optimal discrete sample size can be inferred from the minimizer of f by means of rounding, we think this approximation is justifiable. To this end, we first set

$$d \coloneqq \frac{\alpha\beta}{(\alpha+\beta)^2+(\alpha+\beta+1)} \tag{37.2}$$

We then have for the first derivative of f with respect to n

$$\frac{d}{dn} f(n) = \frac{d}{dn}\left(d\frac{\alpha+\beta}{\alpha+\beta+n} + cn\right) = -d\frac{\alpha+\beta}{(\alpha+\beta+n)^2} + c \tag{37.3}$$

We next define

$$e \coloneqq \alpha + \beta \tag{37.4}$$

and solve the necessary condition for a stationary point of f for a critical value n^{opt}

$$\begin{aligned} \frac{d}{dn} f(n^{opt}) = 0 &\Leftrightarrow -\frac{de}{(e+n)^2} + c = 0 \\ &\Leftrightarrow c(e+n)^2 - de = 0 \\ &\Leftrightarrow n^2 + 2en + \left(e^2 - \frac{de}{c}\right) = 0 \end{aligned} \tag{37.5}$$

with solutions

$$n_{1,2} = -e \pm \sqrt{\frac{de}{c}} = \pm\sqrt{\frac{1}{c}\frac{\alpha\beta(\alpha+\beta)}{(\alpha+\beta)^2+(\alpha+\beta+1)}} - (\alpha+\beta) \tag{37.6}$$

The optimal sample size is thus given by the right-hand side of (37.6), if this is larger than zero, and corresponds to zero else.

Proof of (55)

The KL divergence between two beta distributions distinguished by their parameters α_q, β_q and α_p, β_p, respectively, is given by (see (Liu et al., 2006) for a proof)

$$KL\left(Be\left(\theta; \alpha_q, \beta_q\right) || Be\left(\theta; \alpha_p, \beta_p\right)\right) = \ln\left(\frac{\Gamma(\alpha_q+\beta_q)}{\Gamma(\alpha_q)\Gamma(\beta_q)}\right) - \ln\left(\frac{\Gamma(\alpha_p+\beta_p)}{\Gamma(\alpha_p)\Gamma(\beta_p)}\right) \quad (55.1)$$
$$+\left(\alpha_q - \alpha_p\right)\psi\left(\alpha_q\right) + \left(\beta_q - \beta_p\right)\psi\left(\beta_q\right)$$
$$+(\alpha_p - \alpha_q + \beta_p - \beta_q)\psi\left(\alpha_q + \beta_q\right)$$

from which with $\alpha_q \coloneqq \alpha + r_n, \beta_q \coloneqq \beta + n - r_n, \alpha_p \coloneqq \alpha, \beta_p \coloneqq \beta$ directly follows that

$$KL\left(Be(\theta; \alpha + r_n, \beta + n - r_n) || Be(\theta; \alpha, \beta)\right) \quad (55.2)$$
$$= \ln\left(\frac{\Gamma(\alpha+\beta+n)}{\Gamma(\alpha+r_n)\Gamma(\beta+n-r_n)}\right) - \ln\left(\frac{\Gamma(\alpha+\beta)}{\Gamma(\alpha)\Gamma(\beta)}\right) + r_n\psi(\alpha + r_n) + (n - r_n)\psi(\alpha + r_n) + n\psi(\alpha + \beta + n)$$

Substitution of (55.2) and the functional form of $Be(\theta; \alpha, \beta)$ then directly yields the functional form of h.

References

Bernardo, J.M., and Smith, A. F.M. (1994). Bayesian Theory. JohnWiley and Sons Canada, Ontario, Limited.

Liu, C., Lian, Z., and Han, J. (2006). “How Bayesians debug,” in Proceedings of the Sixth International Conference on Data Mining (Washington, DC: IEEE Computer Society), 382–393.

Pratt, J. W., Raiffa, H., and Schlaifer, R. (1995). Introduction to Statistical Decision Theory. MIT Press, Cambridge.

Matlab Code

```
function niaossdfe

% This function visualizes aspects of the "normative inference approach to
% optimal sample sizes in decisions from experience" as discussed in the
% accompanying manuscript.
%
% Copyright (C) Dirk Ostwald
% -------------------------------------------------------------------------
clc
close all

% -------------------------------------------------------------------------
% ------------------- Inference Approach Simulations ----------------------
% -------------------------------------------------------------------------

% parameters for all simulations
% -------------------------------------------------------------------------
nrep   = 1e3  ; % number of DFE trial simulation repeats
nsim   = 1e2  ; % number of DFE trial simulations

% simulate the "Higher EV" strategy for fsDFE in the gain domain
% -------------------------------------------------------------------------
cumwin_g    = NaN(2,nsim+1,nrep)    ; % initialization of cumulative wins for two decision agents for nrep repeats

% set initial cumulative gain to zero
cumwin_g(:,1,:) = zeros(2,1,nrep);

% cycle over DFE trial simulation repeats
for j = 1:nrep

    % cycle DFE trial simulations
    for i = 1:nsim

        % sample two binary gamble distributions uniformly from
        %             [1,2,...,10] x [1,2,...,10] x ]0,1[
        p_G_AB = [unidrnd(10,[2,2]) rand(2,1)];

        % evaluate the expected values of G_A and G_B
        E_A  = p_G_AB(1,1)*p_G_AB(1,3) + p_G_AB(1,2)*(1-p_G_AB(1,3));
        E_B  = p_G_AB(2,1)*p_G_AB(2,3) + p_G_AB(2,2)*(1-p_G_AB(2,3));

        % simulate decision of a "rational DA"
        if E_A > E_B
            ratio_da = 1;
        else
            ratio_da = 2;
        end

        % simulate decision of a "guessing DA"
        rando_da = unidrnd(2);

        % sample a "final outcomes with economic consequences" from G_A and G_B
        o_AB = [p_G_AB(1,binornd(1,p_G_AB(1,3)) + 1); p_G_AB(2,binornd(1,p_G_AB(2,3)) + 1)];


        % update the cumulative rewards of the "rational" and "random" decision
        % agents
        cumwin_g(1,i+1,j) = cumwin_g(1,i) + o_AB(ratio_da);
        cumwin_g(2,i+1,j) = cumwin_g(2,i) + o_AB(rando_da);

    end
end

% simulate the "Higher EV" strategy for fsDFE in the gain/loss domain
% -------------------------------------------------------------------------
cumwin_gl   = NaN(2,nsim+1,nrep)    ; % initialization of cumulative wins for two decision agents for nrep repeats

% set initial cumulative gain to zero
cumwin_gl(:,1,:) = zeros(2,1,nrep);

% cycle over DFE trial simulation repeats
for j = 1:nrep

    % cycle DFE trial simulations
    for i = 1:nsim

        % sample one binary gamble distribution uniformly from
        %             [1,2,...,10] x [1,2,...,10] x ]0,1[
        % and another binary gamble distribution uinformly from
        %         [-10,-9,...,-1] x [-10,-9,...,-1] x ]0,1[
        p_G_AB = [[unidrnd(10,[1,2]); -unidrnd(10,[1,2])] rand(2,1)];

        % evaluate the expected values of G_A and G_B
        E_A  = p_G_AB(1,1)*p_G_AB(1,3) + p_G_AB(1,2)*(1-p_G_AB(1,3));
        E_B  = p_G_AB(2,1)*p_G_AB(2,3) + p_G_AB(2,2)*(1-p_G_AB(2,3));

        % simulate decision of a "rational DA"
        if E_A > E_B
            ratio_da = 1;
        else
            ratio_da = 2;
        end

        % simulate decision of a "guessing DA"
        rando_da = unidrnd(2);

        % sample a "final outcomes with economic consequences" from G_A and G_B
        o_AB = [p_G_AB(1,binornd(1,p_G_AB(1,3)) + 1); p_G_AB(2,binornd(1,p_G_AB(2,3)) + 1)];

        % update the cumulative rewards of the "rational" and "random" decision
        % agents
        cumwin_gl(1,i+1,j) = cumwin_gl(1,i) + o_AB(ratio_da);
        cumwin_gl(2,i+1,j) = cumwin_gl(2,i) + o_AB(rando_da);

    end
end

% simulate the "Higher EV" strategy for fsDFE in the random gain/loss domain
% -------------------------------------------------------------------------
cumwin_rgl  = NaN(2,nsim+1,nrep)    ; % initialization of cumulative wins for two decision agents for nrep repeats

% set initial cumulative gain to zero
cumwin_rgl(:,1,:) = zeros(2,1,nrep);

% cycle over DFE trial simulation repeats
for j = 1:nrep

    % cycle DFE trial simulations
    for i = 1:nsim

        % sample two binary gamble distributions uniformly from
        %          [-10,-9...,9,10] x [-10,-9...,9,10] x ]0,1[
        o       = -10:10         ; % possible outcomes
        n_o     = length(o)      ; % number of possible outcomes

        p_G_AB = [o(unidrnd(n_o)) o(unidrnd(n_o)) rand; ...
                  o(unidrnd(n_o)) o(unidrnd(n_o)) rand];

        % evaluate the expected values of G_A and G_B
        E_A  = p_G_AB(1,1)*p_G_AB(1,3) + p_G_AB(1,2)*(1-p_G_AB(1,3));
        E_B  = p_G_AB(2,1)*p_G_AB(2,3) + p_G_AB(2,2)*(1-p_G_AB(2,3));

        % simulate decision of a "rational DA"
        if E_A > E_B
            ratio_da = 1;
        else
            ratio_da = 2;
        end

        % simulate decision of a "guessing DA"
        rando_da = unidrnd(2);
```

```
        % sample a "final outcomes with economic consequences" from G_A and G_B
        o_AB = [p_G_AB(1,binornd(1,p_G_AB(1,3)) + 1); p_G_AB(2,binornd(1,p_G_AB(2,3)) + 1)];

        % update the cumulative rewards of the "rational" and "random" decision
        % agents
        cumwin_rgl(1,i+1,j) = cumwin_rgl(1,i) + o_AB(ratio_da);
        cumwin_rgl(2,i+1,j) = cumwin_rgl(2,i) + o_AB(rando_da);

    end
end


% simulate the simplified DFEP inference approach
% -------------------------------------------------------------------------
return_dfep   = NaN(2,nsim+1,nrep); % initialization of returns for two decision agents for nrep repeats

% cycle over DFE trial simulation repeats
for j = 1:nrep

    % cycle DFE trial simulations
    for i = 1:nsim

        % sample a single binary gamble distribution uniformly from
        %         [-10,-9,...,0,...,9,10] x ]0,1[
        o       = -10:10                              ; % possible outcomes
        n_o     = length(o)                           ; % number of possible outcomes
        p_G     = [o(unidrnd(n_o)) o(unidrnd(n_o)) rand];

        % evaluate the expected values of G_A and G_B
        E  = p_G(1)*p_G(3) + p_G(2)*(1-p_G(3));

        % simulate decision of a "rational DA"
        if E > 0
            ratio_da = 1;
        else
            ratio_da = 2;
        end

        % simulate decision of a "guessing DA"
        rando_da = unidrnd(2);

        % sample a "final outcomes with economic consequences" from G
        o_G = p_G(1,binornd(1,p_G(3)) + 1);

        % update the cumulative rewards
        if ratio_da == 1
            return_dfep(1,i,j) = o_G;
        else
            return_dfep(1,i,j) = 0;
        end

        if rando_da == 1
            return_dfep(2,i,j) = o_G;
        else
            return_dfep(2,i,j) = 0;
        end

    end
end


% visualiation
h = figure;
set(h, 'Color', [1 1 1])
subplot(3,4,1)
hold on
plot(0:nsim, mean(cumwin_g(1,:,:),3), 'b', 'LineWidth' , 2)
plot(0:nsim, mean(cumwin_g(2,:,:),3), 'r','LineWidth' , 2)
plot(0:nsim, mean(cumwin_g(1,:,:),3) - mean(cumwin_g(2,:,:),3) , 'k','LineWidth' , 2)
legend('Higher EV', 'Guess', 'Higher EV - Guess', 'Location', 'NorthWest')
set(gca, 'FontName', 'Times New Roman', 'FontSize', 11)
xlabel('DFE Problem')
ylabel('Cumulative Return')
ylim([-100 700])

subplot(3,4,2)
hold on
plot(0:nsim, mean(cumwin_rgl(1,:,:),3), 'b', 'LineWidth' , 2)
plot(0:nsim, mean(cumwin_rgl(2,:,:),3), 'r', 'LineWidth' , 2)
plot(0:nsim, mean(cumwin_rgl(1,:,:),3) - mean(cumwin_rgl(2,:,:),3) , 'k','LineWidth' , 2)
legend('Higher EV', 'Guess', 'Higher EV - Guess', 'Location', 'NorthWest')
set(gca, 'FontName', 'Times New Roman', 'FontSize', 11)
ylabel('Cumulative Return')
xlabel('DFE Problem')
ylim([-100 700])

subplot(3,4,3)
hold on
plot(0:nsim, mean(cumwin_gl(1,:,:),3), 'b', 'LineWidth' , 2)
plot(0:nsim, mean(cumwin_gl(2,:,:),3), 'r','LineWidth' , 2)
plot(0:nsim, mean(cumwin_gl(1,:,:),3) - mean(cumwin_gl(2,:,:),3) , 'k','LineWidth' , 2)
legend('Higher EV', 'Guess', 'Higher EV - Guess', 'Location', 'NorthWest')
set(gca, 'FontName', 'Times New Roman', 'FontSize', 11)
ylabel('Cumulative Return')
xlabel('DFE Problem')
ylim([-100 700])

subplot(3,4,4)
hold on
plot(0:nsim, cumsum(mean(return_dfep(1,:,:),3)), 'b', 'LineWidth' , 2)
plot(0:nsim, cumsum(mean(return_dfep(2,:,:),3)), 'r', 'LineWidth' , 2)
plot(0:nsim, cumsum(mean(return_dfep(1,:,:),3)) - cumsum(mean(return_dfep(2,:,:),3)), 'k', 'LineWidth' , 2)
legend('Positive EV', 'Guess', 'Positive EV - Guess', 'Location', 'NorthWest')
set(gca, 'FontName', 'Times New Roman', 'FontSize', 11)
xlabel('DFE Problem')
ylabel('Cumulative Return')
ylim([-10 120])

% -------------------------------------------------------------------------
% -- Conceptual figure for point and probabilistic interval estimation ----
% -------------------------------------------------------------------------
% parameter space: state/action space
theta = linspace(0,1,1e3);

h = figure;
set(h, 'Color', [1 1 1])
subplot(2,2,1)
hold on
plot(theta, zeros(1,length(theta))     , 'k', 'LineWidth', 1)
plot(theta, zeros(1,length(theta))-0.05, 'b', 'LineWidth', 1)
plot(0.56,    0, 'ko', 'MarkerFaceColor', 'k')
plot(0.60,-0.05, 'bo', 'MarkerFaceColor', 'b')
ylim([-1 1])
box off
axis off

subplot(2,2,2)
hold on
plot(theta, zeros(1,length(theta))     , 'k', 'LineWidth', 1)
plot(theta, zeros(1,length(theta))-0.05, 'b', 'LineWidth', 1)
plot(theta,0.2*pdf('beta', theta, 8, 4 ), 'k', 'LineWidth', 1)
plot(0.60,-0.05, 'bo', 'MarkerFaceColor', 'b')
ylim([-1 1])
box off
axis off

% -------------------------------------------------------------------------
% ---------------------- Parameter Point Estimation -----------------------
% -------------------------------------------------------------------------

% visualize the probabilistic model
% -------------------------------------------------------------------------
b_para      = [[.5 .5]; [10 10]; [20 10]]           ; % beta distribution parameters
p_res       = 1e2                                   ; % resolution of the support set for parameter p
p           = 1/(2*p_res):(1/p_res):(1-1/(2*p_res)) ; % support set of parameter p
```

```matlab
% initialize figure
h = figure;
set(h, 'Color', [1 1 1])

% subplot index
idx = 1;

% cycle over prior distributions
for i = 1:size(b_para,1)

    df_p        = pdf('beta', p, b_para(i,1), b_para(i,2))      ; % probability density function
    mf_p        = (1/p_res)*df_p                                ; % convert pdf to pmf

    % cycle over sample sizes n
    for n = [5 10 30]

        r_n           = 0:n                                      ; % support set of number of success r
        mf_p_k      = NaN(length(p),length(r_n))                 ; % joint distribution over p and r array

        % cycle over marginal values of p and assemble joint pmf
        for j = 1:length(p)

            % evaluate scaled binomial mass function
            mf_p_k(j,:) = mf_p(j).*pdf('Binomial', r_n, n, p(j));

        end

        % visualize the distributions
        subplot(3,3,idx)
        imagesc(r_n,p,mf_p_k)
        idx = idx + 1;
        title(['\alpha = ' num2str(b_para(i,1)),', \beta = ' num2str(b_para(i,2)), ', n = ' num2str(n)], 'FontSize', 24, 'FontName', 'Times New Roman')
        xlabel('r_n', 'FontSize', 24, 'FontName', 'Times New Roman')
        ylabel('\theta', 'Rotation', 0, 'FontSize', 24, 'FontName', 'Times New Roman')
        if n < 30
            set(gca, 'FontSize', 16, 'FontName', 'Times New Roman', 'LineWidth', 2, 'xtick', [0:n])
        else
            set(gca, 'FontSize', 16, 'FontName', 'Times New Roman', 'LineWidth', 2)
        end
    end
end

% visualize the expectation and variance of a beta distribution as a
% function of its parameters
% -------------------------------------------------------------------------
% distribution parameter space
max_p = 100;
min_p = 1e-2;
res_p = 1e3;
alpha = linspace(min_p, max_p, res_p);
beta  = linspace(min_p, max_p, res_p);

% initialize expectation and variance arrays
beta_exp = NaN(length(alpha), length(beta));
beta_var = NaN(length(alpha), length(beta));

% cycle over beta parameter settings
for i = 1:length(alpha)
    for j = 1:length(beta)

        % evaluate expectation
        beta_exp(i,j) = alpha(i)/(alpha(i) + beta(j));

        % evaluate variance
        beta_var(i,j) = ((alpha(i)*beta(j))/((alpha(i) + beta(j))^2*(alpha(i)+beta(j)+1)));

    end
end

% visualize
h = figure;
set(h, 'Color', [1 1 1])
subplot(1,2,1)
imagesc(beta, alpha, beta_exp)
xlabel('\beta', 'FontSize', 25)
ylabel('\alpha', 'Rotation', 0, 'FontSize', 25)
title('Beta Distribution Expectation' ,'FontName', 'Times New Roman', 'FontSize', 20)
axis square
set(gca, 'YDir', 'Normal', 'FontName', 'Times New Roman', 'FontSize', 20)
colorbar('FontName', 'Times New Roman', 'FontSize', 16)

subplot(1,2,2)
imagesc(beta, alpha, sqrt(beta_var), [0 0.1])
xlabel('\beta', 'FontSize', 25)
ylabel('\alpha', 'Rotation', 0, 'FontSize', 25)
title('Beta Distribution Standard Deviation' ,'FontName', 'Times New Roman', 'FontSize', 20)
axis square
set(gca, 'YDir', 'Normal', 'FontName', 'Times New Roman', 'FontSize', 20)
colorbar('FontName', 'Times New Roman', 'FontSize', 16)

% visualize the posterior terminal opportunity loss
% -------------------------------------------------------------------------
% action space
theta_hat       = linspace(0,1,1e3);

% visualize poster terminal opportunity loss as function r_n
n               = 4;
r_n             = 0:n;
alpha_beta      = [0.5 0.5; 4 2; 20 10];

% initialize posterior terminal opportunity loss
PTOL        = NaN(length(r_n), length(theta_hat), size(alpha_beta,1));
theta_opt   = NaN(length(r_n), size(alpha_beta,1));

% cycle over prior parameters
for p = 1:size(alpha_beta,1)

    % prior parameters
    alpha = alpha_beta(p,1);
    beta  = alpha_beta(p,2);

    % cycle over possible outcomes
    for k = 1:length(r_n)

        % evaluate posterior terminal opportunity loss
        PTOL(k,:,p) = ((alpha + r_n(k))*(beta + n - r_n(k)))/(((alpha + beta + n)^2)*(alpha + beta + n + 1)) + (((alpha + r_n(k))/(alpha + beta + n)) - theta_hat).^2;

        % evaluate optimal act
        theta_opt(k,p) = (alpha + r_n(k))/(alpha + beta + n);

    end
end

% visualize
h = figure;
set(h, 'Color', [1 1 1])
colors = {'r', 'm', 'b', 'c', 'y'};

for p = 1:3
    subplot(1,4,p)
    hold on
    for k = 1:length(r_n)
        plot(theta_hat, PTOL(k,:,p), 'Color', colors{k})
        plot(theta_opt(k,p), 0, 'ko', 'MarkerFaceColor', colors{k})
    end
    axis square
    set(gca, 'YDir', 'Normal', 'FontName', 'Times New Roman', 'FontSize', 20)
    ylim([0 .7])
    if p == 3
            legend('r_n = 0','r_n = 1','r_n = 2','r_n = 3', 'r_n = 4')
    end

    title(['\alpha  = ', num2str(alpha_beta(p,1)), ', \beta = ' num2str(alpha_beta(p,1))])
    xlabel('$\hat{\theta}$','interpreter', 'Latex')
    set(gca, 'YDir', 'Normal', 'FontName', 'Times New Roman', 'FontSize', 20)
```

```
end

% visualize minimial posterior terminal opportunity loss for fixed r_n and n...
n   = 16;
r_n = 8;

% ... as function of alpha and beta
alpha = linspace(1e-2,100,1e3);
beta  = linspace(1e-2,100,1e3);

% minimal posterior terminal opportunity loss initialization
MPTOL = NaN(length(alpha), length(beta));

% evaluate the p.t.o.l
for a = 1:length(alpha)
    for b = 1:length(beta)

        % evaluate
        MPTOL(a,b) = ((alpha(a) + r_n)*(beta(b) + n - r_n))/(((alpha(a) + beta(b) + n)^2)*(alpha(a) + beta(b) + n + 1));

    end
end

subplot(1,4,4)
imagesc(beta, alpha, MPTOL)
xlabel('\beta', 'FontSize', 20)
ylabel('\alpha', 'Rotation', 0, 'FontSize', 20)
axis square
set(gca, 'YDir', 'Normal', 'FontName', 'Times New Roman', 'FontSize', 20)

% visualize the expected maximal terminal utility loss
% -------------------------------------------------------------------------
% range of sample sizes considered
n          = linspace(0,30,1e3);

% prior parameters
alpha_beta = [1 1; 2 1; 9 5];

% sampling cost constant
c          = 1e-3;

% intialize expected minimum posterior opportunity loss
EMTOL     = NaN(size(alpha_beta,1),length(n));

% intialize expected minimum posterior opportunity loss + sampling cost
NEMTOL     = NaN(size(alpha_beta,1),length(n));

% initialize optimal sample size
n_opt      = NaN(size(alpha_beta,1));

% cycle over prior parameters
for p = 1:size(alpha_beta,1)

    % prior parameters
    alpha       = alpha_beta(p,1);
    beta        = alpha_beta(p,2);

    % evaluate expected minimum posterior opportunity loss
    EMTOL(p,:)  = (n./(alpha + beta + n)).*((alpha*beta)/(((alpha + beta)^2)*(alpha + beta + 1)));
    NEMTOL(p,:) = (n./(alpha + beta + n)).*((alpha*beta)/(((alpha + beta)^2)*(alpha + beta + 1)))  - c.*n;
    n_opt(p)    =  sqrt((1/c)*((alpha*beta*(alpha + beta))/((alpha + beta)^2*(alpha + beta + 1)))) - (alpha + beta) ;
    if n_opt(p) < 0
        n_opt(p) = 0;
    end


end

% visualize
h = figure;
set(h, 'Color', [1 1 1])
subplot(1,3,1)
colors = {'r', 'm', 'b'};

% create legend
for p  = 1:size(alpha_beta,1)
    hold on
    plot(n, NEMTOL(p,:), colors{p}, 'LineWidth', 2)
    plot(n, EMTOL(p,:) , colors{p}, 'LineStyle', '--', 'LineWidth', 2)
    plot(n, c.*n, 'k', 'LineWidth', 2)
    plot(n_opt(p), -0.02, 'ko', 'MarkerFaceColor', colors{p}, 'MarkerSize', 12)
end
xlim([0 30])

% legend(['h(n),  \alpha = ' num2str(alpha_beta(1,1),2), ', \beta  = ' num2str(alpha_beta(1,2),2)] , ...
%        ['h(n),  \alpha = ' num2str(alpha_beta(2,1),2), ', \beta  = ' num2str(alpha_beta(2,2),2)] , ...
%        ['h(n),  \alpha = ' num2str(alpha_beta(3,1),2), ', \beta  = ' num2str(alpha_beta(3,2),2)])

xlabel('n','FontName', 'Times New Roman', 'LineWidth', 2, 'FontSize', 24)
set(gca, 'YDir', 'Normal', 'FontName', 'Times New Roman', 'LineWidth', 2, 'FontSize', 24)
axis square


% visualize the optimal sample size
% -------------------------------------------------------------------------
% prior distribution space
max_p = 30;
min_p = 1e-2;
res_p = 1e3;
alpha = linspace(min_p, max_p, res_p);
beta  = linspace(min_p, max_p, res_p);

% sampling cost constants
c      = [1e-4 1e-3];

% initialize optimal sample size array
n_opt = NaN(length(alpha), length(beta), length(c));

% evaluate optimal sample size as function of alpha, beta, c
for i = 1:length(c)
    for j = 1:length(alpha)
        for k = 1:length(beta)

            % evaluate optimal sample size
            n = sqrt((1/c(i))*((alpha(j)*beta(k)*(alpha(j) + beta(k)))/((alpha(j) + beta(k))^2*(alpha(j) + beta(k) + 1)))) - (alpha(j) + beta(k));

            if n > 0
                n_opt(j,k,i) = n;
            else
                n_opt(j,k,i) = 0;
            end
        end
    end
end

% visualize
for i = 1:length(c)
    subplot(1,3,i+1)
    imagesc(beta, alpha, n_opt(:,:,i))
    set(gca, 'YDir', 'Normal', 'FontName', 'Times New Roman', 'LineWidth', 2, 'FontSize', 16)
    colorbar('Location', 'North', 'Xcolor', [1 1 1])
    axis square
    xlabel('\beta',  'FontName', 'Times New Roman','FontSize', 24)
    ylabel('\alpha', 'FontName', 'Times New Roman', 'Rotation', 0, 'FontSize', 24)
    title(['n^o^p^t, c = ' num2str(c(i))], 'FontName', 'Times New Roman','FontSize', 24)
end

% -------------------------------------------------------------------------
% ------------ Parameter Probability Interval Estimation ------------------
% -------------------------------------------------------------------------

% visualize the expected information from an experiment
% -------------------------------------------------------------------------
% prior parameters
alpha_beta  = [1 1; 2 1; 9 5];
```

```
% sampling cost constant
c           = 1e-2;

% maximal number of samples considered
max_n       = 100;

% initialize expected information arrays
I_n         = NaN(size(alpha_beta,1),max_n);
NI_n        = NaN(size(alpha_beta,1),max_n);

% initialize optimal sample size
n_opt       = NaN(size(alpha_beta,1),1);

% cycle over prior parameters
for p = 1:size(alpha_beta,1)

    % prior parameters
    alpha       = alpha_beta(p,1);
    beta        = alpha_beta(p,2);

    % cycle over sample sizes
    for n = 1:max_n

        % integration space (possible values of the sufficient statistic)
        r_n         = 0:n;

        % initialize the probability mass function over r_n
        p_r_n       = NaN(1,length(r_n));

        % initialize the posterior alpha and beta parameters
        alpha_post = NaN(length(r_n),1);
        beta_post  = NaN(length(r_n),1);

        % initialize the KL divergences between posterior and prior
        KL_beta     = NaN(length(r_n),1);

        % cycle over possible values of the sufficient statistic
        for k = 1:length(r_n)

            % evaluate the probability of r_n = k under Bb(r_n;alpha,beta,n)
            p_r_n(k)        = pmf_binomial_beta(r_n(k),alpha,beta,n);

            % evaluate the posterior parameters for r_n = k
            alpha_post(k)   = alpha + r_n(k);
            beta_post(k)    = beta + n - r_n(k);

            % evaluate the KL divergence between the posterior and prior for r_n = k
            KL_beta(k)      = kl_beta(alpha_post(k), beta_post(k),alpha, beta);

        end


        % integrate
        I_n(p,n)  = p_r_n*KL_beta;

        % add sampling cost
        NI_n(p,n) = I_n(p,n) - c*n;

    end

    % determine optimal sample size by algorithmic maximization
    [max_NI_n,n_max] = max(NI_n(p,:), [],2);
    n_opt(p)         = n_max;

end

% visualize
h = figure;
set(h, 'Color', [1 1 1])
subplot(1,3,1)
colors      = {'r', 'm', 'b'};
lo_lim_y    = -1;
for p = 1:3
    hold on
    plot(1:max_n, NI_n(p,:), colors{p}, 'LineStyle', '-', 'LineWidth', 2)
    plot(1:max_n, I_n(p,:) , colors{p}, 'LineStyle', '--', 'LineWidth', 2)
    plot(1:max_n, c.*(1:max_n), 'k', 'LineWidth', 2)
    plot(n_opt(p), lo_lim_y, 'ko', 'MarkerFaceColor', colors{p}, 'MarkerSize', 12)
end

% legend(['h(n),  \alpha = ' num2str(alpha_beta(1,1),2), ', \beta  = ' num2str(alpha_beta(1,2),2)] , ...
%        ['h(n),  \alpha = ' num2str(alpha_beta(2,1),2), ',  \beta  = ' num2str(alpha_beta(2,2),2)] , ...
%        ['h(n),  \alpha = ' num2str(alpha_beta(3,1),2), ',  \beta  = ' num2str(alpha_beta(3,2),2)])

ylim([lo_lim_y 2.5])
xlabel('n','FontName', 'Times New Roman', 'LineWidth', 2, 'FontSize', 24)
set(gca, 'YDir', 'Normal', 'FontName', 'Times New Roman', 'LineWidth', 2, 'FontSize', 24)
axis square

% visualize the optimal sample size
% -------------------------------------------------------------------------
%prior distribution space
max_p = 30;
min_p = 1e-2;
res_p = 50;
alpha = linspace(min_p, max_p, res_p);
beta  = linspace(min_p, max_p, res_p);

% sampling cost constants
c       = [5e-3 1e-2];

% initialize optimal sample size array
n_opt   = NaN(length(alpha), length(beta), length(c));

% evaluate optimal sample size as function of alpha, beta, c
for i = 1:length(c)
    for j = 1:length(alpha)

        % it takes some time
        disp(['Evaluating alpha value #' num2str(j) ' of ' num2str(res_p)])

        for h = 1:length(beta)


            % initialize integrated expected information gain and sampling cost function
            n_opt_abc   =  0;   % alpha, beta, c specific optimal sample size
            NI_n_0      =  0;  % I(n) + c*n
            NI_n_1      =  0;  % I(n+1) + c*(n+1)


            % recursively determine optimal sample size
            while (NI_n_1 >= 0) && (NI_n_1 >= NI_n_0)

                % evaluate current sample size of interest
                n           = n_opt_abc + 1;

                % integration space (possible values of the sufficient statistic)
                r_n         = 0:n;

                % initialize the probability mass function over r_n
                p_r_n       = NaN(1,length(r_n));

                % initialize the posterior alpha and beta parameters
                alpha_post  = NaN(length(r_n),1);
                beta_post   = NaN(length(r_n),1);

                % initialize the KL divergences between posterior and prior
                KL_beta     = NaN(length(r_n),1);

                % cycle over possible values of the sufficient statistic
                for k = 1:length(r_n)

                    % evaluate the probability of r_n = k under Bb(r_n;alpha,beta,n)
                    p_r_n(k)        = pmf_binomial_beta(r_n(k),alpha(j),beta(h),n);

                    % evaluate the posterior parameters for r_n = k
```

```
                    alpha_post(k)   = alpha(j) + r_n(k);
                    beta_post(k)    = beta(h)  + n - r_n(k);

                    % evaluate the KL divergence between the posterior and prior for r_n = k
                    KL_beta(k)      = kl_beta(alpha_post(k), beta_post(k), alpha(j), beta(h));

                end

                % shift the while comparison criterion
                NI_n_0 = NI_n_1;

                % integrated expected information gain and sampling cost function
                NI_n_1      = p_r_n*KL_beta - c(i)*n;
                n_opt_abc   = n_opt_abc + 1;
            end

            % determine optimal sample size (undo final + 1 in while loop)
            n_opt(j,h,i) = n_opt_abc - 1;
        end
    end
end

% visualize
for i = 1:length(c)
    subplot(1,3,i+1)
    imagesc(beta, alpha, n_opt(:,:,i))
    set(gca, 'YDir', 'Normal', 'FontName', 'Times New Roman', 'LineWidth', 2, 'FontSize', 16)
    colorbar('Location', 'North', 'Xcolor', [1 1 1])
    axis square
    xlabel('\beta',  'FontName', 'Times New Roman','FontSize', 24)
    ylabel('\alpha', 'FontName', 'Times New Roman', 'Rotation', 0, 'FontSize', 24)
    title(['n^o^p^t, c = ' num2str(c(i))], 'FontName', 'Times New Roman','FontSize', 24)
end


% ------------------------------------------------------------------------
% --- Bayesian Bernoulli inference for prior probability mass function ----
% ------------------------------------------------------------------------
% comparison to Beta scenario
n          = 10                                 ; % size of experiment
r_n        = 8                                  ; % observation of interest
alpha      = 2                                  ; % prior beta distribution parameter \alpha
beta       = 2                                  ; % prior beta distribution parameter \beta
dtheta     = 1e-1                               ; % discretization constant for state space
Theta      = 0:dtheta:1                         ; % space of states of the world
xtheta     = Theta(1:end-1) + (diff(Theta)/2)   ; % support set of states

% evaluate prior probability density function
pdf_pri     = pdf('beta', xtheta, alpha, beta);

% convert prior pdf to prior pmf
pmf_pri     = pdf_pri*dtheta;

% evaluate the joint distribution of experimental outcomes and parameter
r_n_all = 0:10;

% initialize joint distribution of parameter and outcomes
pmf_r_n_all_theta = NaN(length(r_n_all),length(xtheta));
for i = 1:length(r_n_all)
    for j = 1:length(xtheta)
        pmf_r_n_all_theta(i,j) = binopdf(r_n_all(i),n,xtheta(j))*pmf_pri(j);
    end
end

% evaluate the posterior distribution for n = 10, r_n = 8
pmf_pos = NaN(length(xtheta),1);

% evaluate numerator of Bayes theorem
for i = 1:length(xtheta)
    pmf_pos(i) = binopdf(r_n,n,xtheta(i))*pmf_pri(i);
end

% evaluate the denominator of Bayes theorem ("partition function")
py      = sum(pmf_pos);

% evaluate the posterior probability mass function
pmf_pos = pmf_pos/py;

% evaluate the marginal distribution of experimental outcomes numerically
pmf_r_n_all = sum(pmf_r_n_all_theta,2);

% evaluate the marginal distribution of experimental outcomes analytically
pmf_r_n_all_ana = NaN(length(r_n_all),1);
for i = 1:length(pmf_r_n_all_ana)
    pmf_r_n_all_anal(i) = pmf_binomial_beta(r_n_all(i),alpha,beta,n);
end

% visualization
h = figure;
set(h, 'Color', [1 1 1])
subplot(1,4,1)
imagesc(xtheta,r_n_all,pmf_r_n_all_theta)
xlabel('\theta', 'FontName', 'Times New Roman', 'LineWidth', 2, 'FontSize', 20)
ylabel('r_n', 'Rotation', 0, 'FontName', 'Times New Roman', 'LineWidth', 2, 'FontSize', 20)
title(['PMF p_1_0(r_n,\theta), \alpha = ' num2str(alpha) ', \beta = ' num2str(beta)], 'FontName', 'Times New Roman', 'LineWidth', 2, 'FontSize', 16)
set(gca, 'FontName', 'Times New Roman', 'LineWidth', 2, 'FontSize', 12)
axis square

subplot(1,4,2)
hold on
stem(xtheta,pmf_pri, 'LineWidth', 2)
plot(0:1e-3:1, pdf('beta',0:1e-3:1, alpha, beta)*dtheta, 'r', 'LineWidth', 2)
set(gca, 'FontName', 'Times New Roman', 'LineWidth', 2, 'FontSize', 12)
xlabel('\theta', 'FontName', 'Times New Roman', 'LineWidth', 2, 'FontSize', 20)
title('p(\theta)', 'FontName', 'Times New Roman', 'LineWidth', 2, 'FontSize', 20)
legend('PMF', 'PDF \cdot d\theta', 'Location', 'NorthWest')
axis square

subplot(1,4,3)
hold on
stem(xtheta,pmf_pos, 'LineWidth', 2)
plot(0:1e-3:1, pdf('beta',0:1e-3:1, alpha + r_n, beta + n - r_n)*dtheta, 'r', 'LineWidth', 2)
title(['p_1_0(\theta|r_n = ' num2str(r_n) ')'], 'FontName', 'Times New Roman', 'LineWidth', 2, 'FontSize', 20)
set(gca, 'FontName', 'Times New Roman', 'LineWidth', 2, 'FontSize', 12)
xlabel('\theta', 'FontName', 'Times New Roman', 'LineWidth', 2, 'FontSize', 20)
legend('PMF', 'PDF \cdot d\theta', 'Location', 'NorthWest')
axis square

subplot(1,4,4)
hold on
bar(r_n_all, pmf_r_n_all_anal, 'r', 'LineWidth', 2)
stem(r_n_all,pmf_r_n_all , 'LineWidth', 2)
set(gca, 'FontName', 'Times New Roman', 'LineWidth', 2, 'FontSize', 12)
title('p(r_n)', 'FontName', 'Times New Roman', 'LineWidth', 2, 'FontSize', 20)
xlim([-1 n+1])
xlabel('r_n', 'FontName', 'Times New Roman', 'LineWidth', 2, 'FontSize', 20)
legend('Bb(r_n;\alpha,\beta,n)', '\Sigma', 'Location', 'SouthWest')
axis square

% ------------------------------------------------------------------------
% --- Numerical replication of analytic Beta-Binomial results (1) ---------
% ------------------------------------------------------------------------
% comparison to Beta scenario for a single sample size (experiment)
alpha      = 9                                  ; % prior beta distribution parameter \alpha
beta       = 5                                  ; % prior beta distribution parameter \beta
n          = 20                                 ; % sample size considered
dtheta     = 1e-1                               ; % discretization constant for state space
Theta      = 0:dtheta:1                         ; % space of states of the world
xtheta     = Theta(1:end-1) + (diff(Theta)/2)   ; % support set of states

% numerical approach
% ------------------------------------------------------------------------
% evaluate prior probability density function
pdf_pri         = pdf('beta', xtheta, alpha, beta);

% convert prior pdf to prior pmf
```

```
pmf_pri         = pdf_pri*dtheta;

% define action (= point parameter estimator) space
theta_hat_all   = xtheta;

% define outcomes
r_n_all = 0:n;

% initialize the function g(e = n_all(e),z) (eq. (9))
g_e_z_numeric      = NaN(1, length(r_n_all));
g_e_z_anlytic      = NaN(1, length(r_n_all));

% initialize joint distribution of parameter and outcomes
pmf_r_n_all_theta   = NaN(length(r_n_all),length(xtheta));

% initialize analytical marginal distribution over outcomes
pmf_r_n_all_anlytic = NaN(length(xtheta),1);

% initialize the function f(e,z,a) (eq. (8))
f_e_z_a_numeric = NaN(length(r_n_all), length(xtheta));
f_e_z_a_anlytic = NaN(length(r_n_all), length(xtheta));

% cycle over outcomes
% ----------------------------------------------------------------------
for z = 1:length(r_n_all)

    % evaluate the joint distribution marginal outcome distribution p_n(r_n,s)
    for s = 1:length(xtheta)
        pmf_r_n_all_theta(z,s) = binopdf(r_n_all(z),n,xtheta(s))*pmf_pri(s);
    end

    % cycle over actions
    % ------------------------------------------------------------------
    for a = 1:length(theta_hat_all)

        % evaluate the loss function for the current action
        % ------------------------------------------------------------------
        l_a_s = ((xtheta - theta_hat_all(a)).^2)';

        % perform  numerical Bayesian inference
        % ------------------------------------------------------------------
        % evaluate the outcome dependent posterior distribution
        pmf_pos = NaN(length(xtheta),1);

        % cycle over parameters and evaluate numerator of Bayes theorem
        for s = 1:length(xtheta)
            pmf_pos(s) = binopdf(r_n_all(z), n, xtheta(s))*pmf_pri(s);
        end

        % evaluate the denominator of Bayes theorem ("partition function")
        py = sum(pmf_pos);

        % evaluate the posterior probability mass function
        pmf_pos = pmf_pos/py;

        % INNER INTEGRATION OVER STATES
        % evaluate the action, outcome, experiment expected posterior loss
        f_e_z_a_numeric(z,a) = pmf_pos'*l_a_s;
        f_e_z_a_anlytic(z,a) = (((alpha + r_n_all(z))*(beta + n - r_n_all(z)))/(((alpha + beta + n)^2)*(alpha + beta + n + 1))) + ((alpha + r_n_all(z))/(alpha + beta + n) - theta_hat_all(a))^2;


    end

    % INNER MINIMIZATION/MAXIMIZATION OVER ACTION
    % find the minimum posterior loss
    % ------------------------------------------------------------------
    g_e_z_numeric(z)  = min(f_e_z_a_numeric(z,:),[],2);
    g_e_z_anlytic(z)  = (((alpha + r_n_all(z))*(beta + n - r_n_all(z)))/(((alpha + beta + n)^2)*(alpha + beta + n + 1)));

    % analytical evaluation of the marginal distribution over outcomes
    pmf_r_n_all_anlytic(z) = pmf_binomial_beta(r_n_all(z),alpha,beta,n);

end

% evaluation of the marginal distribution
pmf_r_n_all_numeric = sum(pmf_r_n_all_theta,2);


% visualization
h = figure;
set(h, 'Color', [1 1 1])
subplot(1,4,1)
hold on
plot(theta_hat_all, f_e_z_a_anlytic(10,:), 'r:o', 'LineWidth' , 4)
plot(theta_hat_all, f_e_z_a_numeric(10,:), 'bo-' , 'LineWidth', 2)
title('Expected Posterior Loss', 'FontName', 'Times New Roman', 'FontSize', 20)
xlabel('$\hat{\theta}$','interpreter', 'Latex', 'FontSize', 20)
legend('Analytic Solution', 'Numeric Solution')
set(gca, 'FontName', 'Times New Roman', 'FontSize', 20, 'LineWidth', 2)
axis square

subplot(1,4,2)
hold on
plot(r_n_all, g_e_z_anlytic, 'r:o', 'LineWidth', 4)
plot(r_n_all, g_e_z_numeric, 'bo-' , 'LineWidth', 2)
ylim([0 0.02])
xlim([r_n_all(1) r_n_all(end)])
xlabel('r_n', 'FontName', 'Times New Roman', 'FontSize', 20)
set(gca, 'FontName', 'Times New Roman', 'FontSize', 20, 'LineWidth', 2)
title('Min Expected Posterior Loss', 'FontName', 'Times New Roman', 'FontSize', 20)
axis square

subplot(1,4,3)
hold on
stem(r_n_all, pmf_r_n_all_anlytic, 'r:', 'LineWidth', 4)
stem(r_n_all, pmf_r_n_all_numeric, 'b' , 'LineWidth', 2)
xlim([r_n_all(1) r_n_all(end)])
xlabel('r_n', 'FontName', 'Times New Roman', 'FontSize', 20)
set(gca, 'FontName', 'Times New Roman', 'FontSize', 20, 'LineWidth', 2)
title('Marginal Outcome Distribution', 'FontName', 'Times New Roman', 'FontSize', 20)
axis square

% ----------------------------------------------------------------------
% --- Numerical replication of analytic Beta-Binomial results (2) ---------
% ----------------------------------------------------------------------
alpha_beta = [1 1; 2 1; 9 5]                    ; % prior parameters
n_max      = 30                                 ; % sample size considered
c          = 1e-3                               ; % sampling cost constant
dtheta     = 1e-1                               ; % discretization constant for state space
Theta      = 0:dtheta:1                         ; % space of states of the world
xtheta     = Theta(1:end-1) + (diff(Theta)/2)   ; % support set of states

% initalize analytic and numerical h functions
NEMOV      = NaN(size(alpha_beta,1),n_max + 1);
h_e        = NaN(size(alpha_beta,1),n_max + 1);

% initialize optimal sample sizes
n_opt      = NaN(size(alpha_beta,1),1);

% range of sample sizes considered
n          = 0:n_max;

% cycle over prior parameters
for p = 1:size(alpha_beta,1)

    alpha      = alpha_beta(p,1)  ; % prior beta distribution parameter \alpha
    beta       = alpha_beta(p,2)  ; % prior beta distribution parameter \beta

    % analytical approach
    % ------------------------------------------------------------------
    % evaluate expected minimum posterior opportunity loss + sampling cost and optimal sample size
    NEMOV(p,:)      = (n./(alpha + beta + n)).*((alpha*beta)/(((alpha + beta)^2)*(alpha + beta + 1)))  - c.*n;

    % prior variance
    var_theta       = (alpha*beta)/((alpha + beta)^2*(alpha + beta + 1));
```

```
    % numerical approach
    % ------------------------------------------------------------------------
    % evaluate prior probability density function
    pdf_pri         = pdf('beta', xtheta, alpha, beta);

    % convert prior pdf to prior pmf
    pmf_pri         = pdf_pri*dtheta;

    % define action (= point parameter estimator) space
    theta_hat_all   = xtheta;

    % define sample sizes of interest
    n_all           = 0:n_max;

    % cycle over size of the experiment
    % ------------------------------------------------------------------------
    for e = 1:length(n_all)

        % inform user
        disp(['Numerically evaluating sample size n = ' num2str(e-1)])

        % cycle over outcomes
        r_n_all = 0:n_all(e);

        % initialize the function g(e = n_all(e),z) (eq. (9))
        g_e_z = NaN(1, length(r_n_all));

        % initialize joint distribution of parameter and outcomes
        pmf_r_n_all_theta = NaN(length(r_n_all),length(xtheta));

        % cycle over outcomes
        % --------------------------------------------------------------------
        for z = 1:length(r_n_all)

            % evaluate the joint distribution marginal outcome distribution p_n(r_n,s)
            for s = 1:length(xtheta)
                pmf_r_n_all_theta(z,s) = binopdf(r_n_all(z),n_all(e),xtheta(s))*pmf_pri(s);
            end

            % initialize the function f(e,z,a) (eq. (8))
            f_e_z_a = NaN(n_all(e), length(r_n_all), length(xtheta));

            % cycle over actions
            % ----------------------------------------------------------------
            for a = 1:length(theta_hat_all)

                % evaluate the loss function for the current action
                % ------------------------------------------------------------
                l_a_s = ((xtheta - theta_hat_all(a)).^2)';

                % perform  numerical Bayesian inference
                % ------------------------------------------------------------
                % evaluate the outcome dependent posterior distribution
                pmf_pos = NaN(length(xtheta),1);

                % cycle over parameters and evaluate numerator of Bayes theorem
                for s = 1:length(xtheta)
                    pmf_pos(s) = binopdf(r_n_all(z),n_all(e), xtheta(s))*pmf_pri(s);
                end

                % evaluate the denominator of Bayes theorem ("partition function")
                py = sum(pmf_pos);

                % evaluate the posterior probability mass function
                pmf_pos = pmf_pos/py;

                % INNER INTEGRATION OVER STATES
                % evaluate the action, outcome, experiment expected posterior loss
                f_e_z_a(e,z,a)            = pmf_pos'*l_a_s;

            end

            % INNER MINIMIZATION/MAXIMIZATION OVER ACTION
            % find the minimum posterior loss
            % ----------------------------------------------------------------
            g_e_z(z)             = min(f_e_z_a(e,z,:),[],3);

        end

        % evaluate the marginal distribution of experimental outcomes numerically
        % --------------------------------------------------------------------
        pmf_r_n_all = sum(pmf_r_n_all_theta,2);


        % OUTER INTEGRATION, SAMPLING COST, AND OFFSET ADDITION
        % --------------------------------------------------------------------
        h_e(p,e)      =  - pmf_r_n_all'*g_e_z' -  c*n_all(e) +  var_theta;

    end

    % OUTER MINIMIZATION/MAXIMIZATION OVER EXPERIMENTS
    [h_e_opt idx_e_opt] = max(h_e(p,:),[],2);

    % optimal sample size
    n_opt(p) = n_all(idx_e_opt);

end

% visualize
subplot(1,4,4)
colors = {'r', 'm', 'b'};
for p  = 1:size(alpha_beta,1)
    hold on
    plot(n, h_e(p,:), colors{p}, 'LineWidth', 2)
end
xlim([0 30])

for p  = 1:size(alpha_beta,1)
    hold on
    plot(n, NEMOV(p,:) , colors{p}, 'LineStyle', '--', 'LineWidth', 2)
    plot(n_opt(p), -0.02, 'ko', 'MarkerFaceColor', colors{p}, 'MarkerSize', 10)
end
xlim([0 30])
ylim([-0.02 0.06])

legend(['h(n),  \alpha = ' num2str(alpha_beta(1,1),2), ', \beta  = ' num2str(alpha_beta(1,2),2)] , ...
       ['h(n),  \alpha = ' num2str(alpha_beta(2,1),2), ', \beta  = ' num2str(alpha_beta(2,2),2)] , ...
       ['h(n),  \alpha = ' num2str(alpha_beta(3,1),2), ', \beta  = ' num2str(alpha_beta(3,2),2)])

xlabel('n','FontName', 'Times New Roman', 'LineWidth', 2, 'FontSize', 20)
set(gca, 'YDir', 'Normal', 'FontName', 'Times New Roman', 'LineWidth', 2, 'FontSize', 20)
axis square

% ------------------------------------------------------------------------
% -------------------- Numerical proof of principle (1) ------------------
% ------------------------------------------------------------------------
n          = 15                                  ; % sample size considered
dtheta     = 1e-1                                ; % discretization constant for state space
Theta      = 0:dtheta:1                          ; % space of states of the world
xtheta     = Theta(1:end-1) + (diff(Theta)/2)    ; % support set of states

% numerical approach
% ------------------------------------------------------------------------
% define prior pmf
pmf_pri         = [.00 .05 .05 .20 .20 .20 .20 .05 .05 .00];

% define action (= point parameter estimator) space
theta_hat_all   = xtheta;

% define outcomes
r_n_all         = 0:n;

% initialize the function g(e = n_all(e),z) (eq. (9))
g_e_z_numeric       = NaN(1, length(r_n_all));

% initialize joint distribution of parameter and outcomes
```

```
pmf_r_n_all_theta   = NaN(length(r_n_all),length(xtheta));

% initialize the function f(e,z,a) (eq. (8))
f_e_z_a_numeric = NaN(length(r_n_all), length(xtheta));

% cycle over outcomes
% ---------------------------------------------------------------------
for z = 1:length(r_n_all)

    % evaluate the joint distribution marginal outcome distribution p_n(r_n,s)
    for s = 1:length(xtheta)
        pmf_r_n_all_theta(z,s) = binopdf(r_n_all(z),n,xtheta(s))*pmf_pri(s);
    end

    % cycle over actions
    % ----------------------------------------------------------------
    for a = 1:length(theta_hat_all)

        % evaluate the terminal utility function for the current action
        % ----------------------------------------------------------------
        u_a_s = NaN(1,length(xtheta));

        % cycle over state space
        for s = 1:length(xtheta)
            if abs(xtheta(s) - theta_hat_all(a)) <= 0.2
                u_a_s(s) = 1;
            else
                u_a_s(s) = 0;
            end
        end

        % save one action dependent utility function for demonstration
        if a == 5
            u_a_s_demo = u_a_s;
        end

        % perform  numerical Bayesian inference
        % -----------------------------------------------------------------
        % evaluate the outcome dependent posterior distribution
        pmf_pos = NaN(length(xtheta),1);

        % cycle over parameters and evaluate numerator of Bayes theorem
        for s = 1:length(xtheta)
            pmf_pos(s) = binopdf(r_n_all(z), n, xtheta(s))*pmf_pri(s);
        end

        % evaluate the denominator of Bayes theorem ("partition function")
        py = sum(pmf_pos);

        % evaluate the posterior probability mass function
        pmf_pos = pmf_pos/py;

        % INNER INTEGRATION OVER STATES
        % evaluate the action, outcome, experiment expected posterior utility
        f_e_z_a_numeric(z,a) = pmf_pos'*u_a_s';

    end

    % INNER MAXIMIZATION OVER ACTION
    % find the maximum posterior utility
    % ----------------------------------------------------------------
    g_e_z_numeric(z)  = max(f_e_z_a_numeric(z,:),[],2);


end

% evaluation of the marginal distribution
pmf_r_n_all_numeric = sum(pmf_r_n_all_theta,2);

% visualization
h = figure;
set(h, 'Color', [1 1 1])

subplot(3,3,1)
hold on
stem(xtheta, pmf_pri, 'LineWidth', 1)
xlabel('\theta', 'FontSize', 14, 'FontName', 'Times New Roman')
title('Prior Distribution', 'FontSize', 14, 'FontName', 'Times New Roman', 'FontWeight', 'Normal')
set(gca, 'FontName', 'Times New Roman', 'FontSize', 14, 'LineWidth', 1)
ylim([0 0.3])

subplot(3,3,2)
hold on
stem(r_n_all, pmf_r_n_all_numeric, 'LineWidth', 1)
xlim([r_n_all(1) r_n_all(end)])
xlabel('r_n', 'FontName', 'Times New Roman', 'FontSize', 14)
set(gca, 'FontName', 'Times New Roman', 'FontSize', 14, 'LineWidth', 1)
title('Marginal Outcome Distribution', 'FontSize', 14, 'FontName', 'Times New Roman', 'FontWeight', 'Normal')

subplot(3,3,3)
hold on
stem(theta_hat_all, u_a_s_demo, 'LineWidth', 1)
xlabel('$\hat{\theta}$','Interpreter', 'Latex', 'FontSize', 14)
title('Terminal Utility', 'FontSize',14, 'FontName', 'Times New Roman', 'FontWeight', 'Normal')
set(gca, 'FontName', 'Times New Roman', 'FontSize', 14, 'LineWidth', 1)
ylim([0 1.5])

subplot(3,3,4)
hold on
stem(theta_hat_all, f_e_z_a_numeric(10,:), 'LineWidth', 1)
title('Expected Terminal Utility', 'FontSize', 14, 'FontName', 'Times New Roman', 'FontWeight', 'Normal')
xlabel('$\hat{\theta}$','interpreter', 'Latex', 'FontSize', 14)
set(gca, 'FontName', 'Times New Roman', 'FontSize', 14, 'LineWidth', 1)

subplot(3,3,5)
hold on
stem(r_n_all, g_e_z_numeric, 'LineWidth', 1)
xlim([r_n_all(1) r_n_all(end)])
xlabel('r_n', 'FontName', 'Times New Roman', 'FontSize', 14)
set(gca, 'FontName', 'Times New Roman', 'FontSize', 14, 'LineWidth', 1)
title('Max Expected Terminal Utility', 'FontName', 'Times New Roman', 'FontSize', 14, 'FontWeight', 'Normal')
ylim([0.9 1.0])


% ----------------------------------------------------------------------
% -------------------- Numerical proof of principle (2) -----------------
% ----------------------------------------------------------------------
n_max      = 30                                 ; % sample sizes considered
dtheta     = 1e-1                               ; % discretization constant for state space
Theta      = 0:dtheta:1                         ; % space of states of the world
xtheta     = Theta(1:end-1) + (diff(Theta)/2)   ; % support set of states
c          = 1e-3                               ; % sampling cost

% numerical approach
% ----------------------------------------------------------------------
% define prior pmf
pmf_pri         = [.00 .05 .05 .20 .20 .20 .20 .05 .05 .00];

% initalize analytic and numerical h functions
h_e             = NaN(1,n_max + 1);

% define action (= point parameter estimator) space
theta_hat_all   = xtheta;

% define sample sizes of interest
n_all           = 0:n_max;

% cycle over size of the experiment
% ----------------------------------------------------------------------
for e = 1:length(n_all)

    % inform user
    disp(['Numerically evaluating sample size n = ' num2str(e-1)])

    % cycle over outcomes
    r_n_all = 0:n_all(e);
```

```
    % initialize the function g(e = n_all(e),z) (eq. (9))
    g_e_z = NaN(1, length(r_n_all));

    % initialize joint distribution of parameter and outcomes
    pmf_r_n_all_theta = NaN(length(r_n_all),length(xtheta));

    % cycle over outcomes
    % -------------------------------------------------------------------
    for z = 1:length(r_n_all)

        % evaluate the joint distribution marginal outcome distribution p_n(r_n,s)
        for s = 1:length(xtheta)
            pmf_r_n_all_theta(z,s) = binopdf(r_n_all(z),n_all(e),xtheta(s))*pmf_pri(s);
        end

        % initialize the function f(e,z,a) (eq. (8))
        f_e_z_a = NaN(n_all(e), length(r_n_all), length(xtheta));

        % cycle over actions
        % ---------------------------------------------------------------
        for a = 1:length(theta_hat_all)

            % evaluate the terminal utility function for the current action
            % ---------------------------------------------------------------
            u_a_s = NaN(1,length(xtheta));

            % cycle over state space
            for s = 1:length(xtheta)
                if abs(xtheta(s) - theta_hat_all(a)) <= 0.2
                    u_a_s(s) = 1;
                else
                    u_a_s(s) = 0;
                end
            end

            % perform  numerical Bayesian inference
            % -----------------------------------------------------------
            % evaluate the outcome dependent posterior distribution
            pmf_pos = NaN(length(xtheta),1);

            % cycle over parameters and evaluate numerator of Bayes theorem
            for s = 1:length(xtheta)
                pmf_pos(s) = binopdf(r_n_all(z),n_all(e), xtheta(s))*pmf_pri(s);
            end

            % evaluate the denominator of Bayes theorem ("partition function")
            py = sum(pmf_pos);

            % evaluate the posterior probability mass function
            pmf_pos = pmf_pos/py;

            % INNER INTEGRATION OVER STATES
            % evaluate the action, outcome, experiment expected posterior loss
            f_e_z_a(e,z,a)            = pmf_pos'*u_a_s';

        end

        % INNER MINIMIZATION/MAXIMIZATION OVER ACTION
        % find the maximum posterior utility
        % ---------------------------------------------------------------
        g_e_z(z)             = max(f_e_z_a(e,z,:),[],3);

    end

    % evaluate the marginal distribution of experimental outcomes numerically
    % -------------------------------------------------------------------
    pmf_r_n_all = sum(pmf_r_n_all_theta,2);


    % OUTER INTEGRATION, SAMPLING COST, AND OFFSET ADDITION
    % -------------------------------------------------------------------
    h_e(e)    = pmf_r_n_all'*g_e_z' - c*n_all(e);

end

% OUTER MAXIMIZATION OVER EXPERIMENTS
[h_e_opt idx_e_opt] = max(h_e,[],2);

% visualize
subplot(3,3,6)
hold on
plot(n_all           , h_e , 'bo-', 'LineWidth', 1)
plot(n_all(idx_e_opt), 0.85, 'ko', 'MarkerFaceColor', 'b', 'MarkerSize', 10)
xlim([0  30])
ylim([0.85  1.00])
title('h(n)', 'FontName', 'Times New Roman', 'FontSize', 14, 'FontWeight', 'Normal')
set(gca, 'FontName', 'Times New Roman', 'FontSize', 14, 'LineWidth', 1)

% -----------------------------------------------------------------------
% -------------------- Numerical proof of principle (3) -----------------
% -----------------------------------------------------------------------
dtheta     = 1e-1                               ; % discretization constant for state space
Theta      = 0:dtheta:1                         ; % space of states of the world
xtheta     = Theta(1:end-1) + (diff(Theta)/2)   ; % support set of states
c          = 1e-3                               ; % sampling cost

% numerical approach
% -----------------------------------------------------------------------
% define prior pmf
pmf_pri         = [.00 .05 .05 .20 .20 .20 .20 .05 .05 .00];

% define action (= point parameter estimator) space
theta_hat_all   = xtheta;

% define sample sizes of interest
n_all           = [14 19 29];

% define number of samples from marginal outcome distribution
nsamp           = 1e2;

% initalize sampled expected utility
u_t             = NaN(nsamp,length(n_all));

% cycle over size of the experiment
% -----------------------------------------------------------------------
for e = 1:length(n_all)

    % inform user
    disp(['Numerically evaluating sample size n = ' num2str(n_all(e))])

    % cycle over outcomes
    r_n_all = 0:n_all(e);

    % initialize the function g(e = n_all(e),z) (eq. (9)) over samples
    g_e_z = NaN(1,nsamp);

    % initialize joint distribution of parameter and outcomes
    pmf_r_n_all_theta = NaN(length(r_n_all),length(xtheta));

    % cycle over outcomes
    % -------------------------------------------------------------------
    for z = 1:length(r_n_all)

        % evaluate the joint distribution marginal outcome distribution p_n(r_n,s)
        for s = 1:length(xtheta)
            pmf_r_n_all_theta(z,s) = binopdf(r_n_all(z),n_all(e),xtheta(s))*pmf_pri(s);
        end

    end

    % evaluate the marginal distribution of experimental outcomes numerically
    % -------------------------------------------------------------------
    pmf_r_n_all = sum(pmf_r_n_all_theta,2);

    % cycle over samples from marginal distribution over outcomes
```

```
    % ----------------------------------------------------------------------
    for i = 1:nsamp

        % sample from (subjective) marginal distribution over outcomes
        % ------------------------------------------------------------------
        r_n = r_n_all(mnrnd(1,pmf_r_n_all) == 1);

        % evaluate outcome dependent expected posterior loss
        % ------------------------------------------------------------------
        % initialize the function f(e,z,a) (eq. (8))
        f_e_z_a = NaN(n_all(e),length(xtheta));

        % cycle over actions
        % ------------------------------------------------------------------
        for a = 1:length(theta_hat_all)

            % evaluate the terminal utility function for the current action
            % --------------------------------------------------------------
            u_a_s = NaN(1,length(xtheta));

            % cycle over state space
            for s = 1:length(xtheta)
                if abs(xtheta(s) - theta_hat_all(a)) <= 0.2
                    u_a_s(s) = 1;
                else
                    u_a_s(s) = 0;
                end
            end

           % perform  numerical Bayesian inference
           % --------------------------------------------------------------
           % evaluate the outcome dependent posterior distribution
           pmf_pos = NaN(length(xtheta),1);

           % cycle over parameters and evaluate numerator of Bayes theorem
           for s = 1:length(xtheta)
                pmf_pos(s) = binopdf(r_n,n_all(e), xtheta(s))*pmf_pri(s);
           end

           % evaluate the denominator of Bayes theorem ("partition function")
           py = sum(pmf_pos);

           % evaluate the posterior probability mass function
           pmf_pos = pmf_pos/py;

           % INNER INTEGRATION OVER STATES
           % evaluate the action, outcome, experiment expected posterior loss
           f_e_z_a(e,a) = pmf_pos'*u_a_s';

        end

        % INNER MINIMIZATION/MAXIMIZATION OVER ACTION
        % find the maximum posterior utility
        % ------------------------------------------------------------------
        g_e_z(i) = max(f_e_z_a(e,:),[],2);

        % evaluate the expected utility
        % ------------------------------------------------------------------
        u_t(i,e) = g_e_z(i) - c*n_all(e);

    end
end

subplot(3,3,7)
hold on
for e = 1:length(n_all)
    plot(1:nsamp, u_t(:,e), 'LineWidth', 1)
end
set(gca, 'FontName', 'Times New Roman', 'FontSize', 14, 'LineWidth',1)
title('Terminal Utility', 'FontName', 'Times New Roman', 'FontSize', 14,'FontWeight', 'Normal')
xlabel('Sample Number', 'FontName', 'Times New Roman', 'FontSize', 14)

subplot(3,3,8:9)
hold on
for e = 1:length(n_all)
    plot(1:nsamp, cumsum(u_t(:,e))./(1:nsamp)' , 'LineWidth', 1)
end
legend(['n = ' num2str(n_all(1))],['n = ' num2str(n_all(2))],['n = ' num2str(n_all(3))], 'Location', 'SouthEast')
title('Terminal Utility (Cumulative Average)', 'FontName', 'Times New Roman', 'FontSize', 14, 'FontWeight', 'Normal')
xlabel('Sample Number', 'FontName', 'Times New Roman', 'FontSize', 14)
set(gca, 'FontName', 'Times New Roman', 'FontSize', 14, 'LineWidth',1)

end

% Subfunctions
% -------------------------------------------------------------------------
function p = pmf_binomial_beta(x,a,b,n)

% This function evaluates the binomial beta probability function.
%
%   Inputs
%           x       : argument of the function
%           a       : alpha parameter
%           b       : beta parameter
%           n       : n parameter
%
%   Output
%           p       : probability of x under binomial-beta
%
% Copyright (C) Dirk Ostwald
% -------------------------------------------------------------------------

% evaluate the normalizing constant
C = gamma(a+b)/(gamma(a)*gamma(b)*gamma(a+b+n));

% evaluate the probability function
warning off
p = C*nchoosek(n,x)*gamma(a+x)*gamma(b+n-x);
warning on

end

function [d] = kl_beta(alpha_q, beta_q, alpha_p, beta_p)

% This function evaluates the KL divergence between a beta distribution B_q
% with parameters alpha_q and beta_q, and a beta distribution B_p with
% parameters alpha_p and beta_p
%
%                     KL(B_q||B_p) = <log B_q/B_p>_B_q
%
%   Inputs
%       alpha_q     : alpha parameter of B_q
%       beta_q      : beta  parameter of B_q
%       alpha_p     : alpha parameter of B_p
%       beta_p      : beta parameter  of B_p
%
%   Ouput
%       d           : KL divergence
%
% Copyright (C) Dirk Ostwald
% -------------------------------------------------------------------------
T1 = log(gamma(alpha_q + beta_q)/(gamma(alpha_q)*gamma(beta_q)));
T2 = - log(gamma(alpha_p + beta_p)/(gamma(alpha_p)*gamma(beta_p)));
T3 = (alpha_q - alpha_p)*psi(alpha_q);
T4 = (beta_q - beta_p)*psi(beta_q);
T5 = (alpha_p - alpha_q + beta_p - beta_q)*psi(alpha_q + beta_q);

d  = T1 + T2 + T3 + T4 + T5;

end
```
